# NGF steers microglia toward a neuroprotective phenotype

**DOI:** 10.1002/glia.23312

**Published:** 2018-02-23

**Authors:** Caterina Rizzi, Alexia Tiberi, Michela Giustizieri, Maria Cristina Marrone, Francesco Gobbo, Nicola Maria Carucci, Giovanni Meli, Ivan Arisi, Mara D'Onofrio, Silvia Marinelli, Simona Capsoni, Antonino Cattaneo

**Affiliations:** ^1^ Bio@SNS Laboratory, Scuola Normale Superiore, Piazza dei Cavalieri 7 Pisa 56126 Italy; ^2^ European Brain Research Institute‐Fondazione Rita Levi Montalcini, Viale Regina Elena 295 Roma 00161, Italy; ^3^ Section of Human Physiology, Department of Biomedical and Specialty Surgical Sciences University of Ferrara, Via Fossato di Mortara 17‐19 Ferrara 44121 Italy

**Keywords:** amyloid, macropinocytosis, microglia, nerve growth factor, neuroinflammation, neuroimmune communication, neuroprotection, neurotrophin

## Abstract

Microglia are the sentinels of the brain but a clear understanding of the factors that modulate their activation in physiological and pathological conditions is still lacking. Here we demonstrate that Nerve Growth Factor (NGF) acts on microglia by steering them toward a neuroprotective and anti‐inflammatory phenotype. We show that microglial cells express functional NGF receptors in vitro and ex vivo. Our transcriptomic analysis reveals how, in primary microglia, NGF treatment leads to a modulation of motility, phagocytosis and degradation pathways. At the functional level, NGF induces an increase in membrane dynamics and macropinocytosis and, in vivo, it activates an outward rectifying current that appears to modulate glutamatergic neurotransmission in nearby neurons. Since microglia are supposed to be a major player in Aβ peptide clearance in the brain, we tested the effects of NGF on its phagocytosis. NGF was shown to promote TrkA‐mediated engulfment of Aβ by microglia, and to enhance its degradation. Additionally, the proinflammatory activation induced by Aβ treatment is counteracted by the concomitant administration of NGF. Moreover, by acting specifically on microglia, NGF protects neurons from the Aβ‐induced loss of dendritic spines and inhibition of long term potentiation. Finally, in an ex‐vivo setup of acute brain slices, we observed a similar increase in Aβ engulfment by microglial cells under the influence of NGF. Our work substantiates a role for NGF in the regulation of microglial homeostatic activities and points toward this neurotrophin as a neuroprotective agent in Aβ accumulation pathologies, via its anti‐inflammatory activity on microglia.

## 1 INTRODUCTION

Microglia are the resident immune cells of the central nervous system (CNS). Beside classic inflammatory activities shared with macrophages, microglia are responsible for brain homeostasis and monitor the brain environment with their ever‐moving processes (Nimmerjahn, Kirchhoff, & Helmchen, 2005; Wolf, Boddeke, & Kettenmann, 2017). They take part in sculpting neuronal circuitries during development (Paolicelli et al., 2011; Wu, Dissing‐Olesen, MacVicar, & Stevens, 2015) and actively participate in activity‐dependent plasticity and learning processes (Parkhurst et al., 2013; Sipe et al., 2016). Microglia have been shown to be key players in the pathogenesis and progression of many neurodegenerative disorders. However, their role—either promoting or preventing pathology—is debated. On one hand, excessive activation of microglia leads to oxidative stress, neuroinflammation, and eventually neuronal death (Block, Zecca, & Hong, 2007). In contrast, the modulation of microglial activation might be harnessed to carry out protective activities in the brain, such as phagocytosis of aggregates, synaptic pruning and formation, and the maintenance of healthy neuronal circuits (Diaz‐Aparicio, Beccari, Abiega, & Sierra, 2016; Keren‐Shaul et al., 2017). Therefore, there is a compelling urgency to find ways to selectively target microglia neuroprotective activities, sparing, or even inhibiting, those features known to be pathological mediators.

The idea of harnessing the CNS immune system—the natural scavengers of the brain—to boost neuroprotection in the brain is intriguing, especially when tackling diseases marked by loss of proteostasis such as Alzheimer's disease (AD; Ardura‐Fabregat et al., 2017). In the search of neuroprotective agents against neurodegeneration, neurotrophins have been historically considered as potential therapeutic candidates, mostly due to their actions on neuronal targets. Microglia themselves are a source of neurotrophins (Elkabes, DiCicco‐Bloom, & Black, 1996; Heese, Hock, & Otten, 1998): microglial‐derived Brain Derived Neurotrophic Factor (BDNF) has been shown to promote synapse formation (Parkhurst et al., 2013). As for Nerve Growth Factor (NGF), this neurotrophin reportedly acts by modulating microglial migratory activity in vitro (De Simone et al., 2007). Macrophages, the peripheral counterparts of microglia, are a target of both mature and pro‐NGF (Williams et al., 2015). However, to what extent NGF might affect physiological microglial functions—and how alterations in this modulation might come into play in neurodegenerative disorders—has not been systematically investigated yet.

Indeed, the main cellular targets of the neurotrophin NGF (Levi‐Montalcini, 1952) in the CNS are considered to be the cholinergic neurons of the basal forebrain (BFCNs; Hefti, 1986). Consistently, interfering with NGF signaling in the adult brain leads to deficits of the cholinergic system (Capsoni et al., 2000; Fagan, Garber, Barbacid, Silos‐Santiago, & Holtzman, 1997; Nagahara et al., 2009; Ruberti et al., 2000). The expression of anti‐NGF antibodies selectively neutralizing mature NGF (Capsoni et al., 2000; Ruberti et al., 2000) or of antibodies neutralizing TrkA (Capsoni et al., 2000; Capsoni, Tiveron, Vignone, Amato, & Cattaneo, 2010) in the adult brain of transgenic mice, determines a progressive comprehensive neurodegeneration, synaptic and behavioral deficits. Changes in NGF homeostasis in the brain, with particular regard to the ratio of NGF to proNGF levels, have also been linked to Alzheimer's disease (Cattaneo & Calissano, 2012). The overall neurodegenerative picture induced by anti‐NGF or anti‐TrkA antibodies in those transgenic models is, however, much broader than what one would expect on the basis of an action of the antibodies exclusively on the BFCNs. Moreover, the loss of NGF‐TrkA signaling “in the CNS”, obtained by conditionally deleting NGF or TrkA genes in CNS cells derived from nestin‐positive cells, has proven not to be sufficient in inducing severe cognitive impairments nor neurodegeneration in mice (Muller et al., 2012).

Altogether, this body of results motivated our search for non‐neuronal targets of NGF in the adult brain. Microglia was a strong candidate, because (1) previous work had suggested that NGF could modulate some aspects of microglial cells in culture (De Simone et al., 2007) and (2) transcriptomic studies in the AD11 mouse model—expressing anti‐NGF—had shown that neuroinflammation is the earliest phenotypic alteration, already at a presymptomatic phase (1 month of age; Capsoni et al., 2011; D'Onofrio et al., 2011).

In this article, we provide now stringent evidence that microglia are target cells for NGF, both in vitro and ex vivo and that the activity carried out by this neurotrophin on microglial cells might result neuroprotective and anti‐inflammatory in the context of Alzheimer's disease.

## 2 MATERIALS AND METHODS

### 2.1 Animals

Adult C57BL/6, Cx3cr1/GFP+/+ mice and B6129 mice were purchased from The Jackson Laboratory (Bar Harbor, ME). Genotyping of CX3CR1‐GFP mice was performed by PCR analysis of tail DNA (IDT 14276: 5′‐GTC TTC ACG TTC GGT CTG GT‐3′, IDT 14277 5′‐CCC AGA CAC TCG TTG TCC TT‐3′, IDT 14278 5′‐CTC CCC CTG AAC CTG AAA C‐3′). All experiments with mice were performed according to the national and international laws for laboratory animal welfare and experimentation (EU directive n. 2010/63/EU and Italian DL n. 26 04/03/2014). Mice were kept under a 12‐hr dark to light cycle, with food and water ad libitum.

### 2.2 Cell cultures

The immortalized BV‐2 murine microglial cell line (Blasi, Barluzzi, Bocchini, Mazzolla, & Bistoni, 1990) was maintained in RPMI (Thermo Fisher Scientific, MA; #11835‐063) medium containing 1% penicillin/streptomycin (Euroclone, MI, Italy; #ECB3001D), 1% Glutamax (Thermo Fisher Scientific; #35050‐038) and 10% fetal bovine serum (FBS; Euroclone; #ECS0180l) in 5% CO_2_ at 37°C.

Primary microglial cells were derived from the brains of B6129 or *Cx3cr1/GFP* ^+/+^ mice at P3–4 as previously described (Butovsky et al., 2014). Cells were maintained in Dulbecco's modified Eagle's medium (DMEM/F12; Thermo Fisher Scientific; #21331‐020) containing 1% penicillin/streptomycin, 1% Glutamax and 10% FBS in 5% CO_2_ pH 7.4 at 37°C. Microglia were separated from the mixed primary glial cultures by mild shaking, they were re‐suspended in DMEM/F12 with 1% penicillin/streptomycin, 1% Glutamax and 10% FBS—*this is the standard culture medium unless otherwise stated*—and plated on the appropriate support 18 hrs before the experiments.

Primary cortical and hippocampal neurons were prepared at P0 as described (Beaudoin et al., 2012). Briefly, animals were decapitated, the brain was rapidly excised and placed into ice‐cold Hanks Buffered Saline Solution (HBSS; Thermo Fisher Scientific, Waltham, MA; #14180046). Hippocampi and cortex were removed and digested for 15 min at 37°C in DMEM‐F12 containing 0.1% of trypsin (Thermo Fisher Scientific). Tissue was transferred in culture medium containing 10% FBS and gently disrupted using a flame‐polished Pasteur pipette. Following centrifugation at 4°C for 8 min at 800 rpm, cells were resuspended in fresh DMEM containing 1% Glutamax, 10% FBS, 2% B27 supplement (Gibco, Waltham, MA; #17504‐044), 6 mg/ml Glucose, 12.5 µM Glutamate, 10 µg/ml Gentamicin (Gibco; #15710‐049) and plated (150,000 cells/coverslip) after proper poly‐D‐lysine coating (Sigma‐Aldrich, St. Louis, MO; #P1024). Cells were kept at 37°C in 5% CO_2_. After 12–24 hr, medium was replaced with Neurobasal A medium (Thermo Fisher Scientific; #10888‐022) containing 2% of B27 supplement, 2.5 µM Glutamax, and 10 µg/ml Gentamicin. The second day 2.5 µM AraC (Sigma‐Aldrich; #C1768) was added to the medium. The experiments were performed at DIV 17–19.

### 2.3 Immunoblot analysis

*NGF signaling*: Primary B6129 microglia were plated in six‐well plates (5 × 10^5^cells/well) in culture medium. Cells were serum‐starved for 16 hr before the start of the treatments, then they were treated for 0, 5, 15, and 30 min with NGF 100 ng/ml and sequentially collected and lysed in ice‐cold RIPA buffer (50 mm Tris‐HCl, pH 7.6, 150 mm NaCl, 1% Igepal, 1 mm EDTA, 1% SDS, 0.5% sodium deoxycholate, 1× protease and phosphatase inhibitor cocktails [Roche, Basel; CH]). After sonication, cells were collected by centrifugation for 15 min at 4°C (13,000 rpm). Protein concentrations of the cell lysates were measured using the Bradford method. Lysates (20 µg) were then separated on a 10% SDS‐PAGE, transferred to a nitrocellulose membrane, and analyzed by Western blotting.

*Phagocytosis of* Aβ: Primary B6129 microglia were first plated in six‐well plates (5 × 10^5^cells/well) in culture medium. They were treated with 1 μM soluble Aβ for 3 hr with or without 100 ng/ml NGF. After collection, they were lysed in ice‐cold RIPA buffer and electrophoresed on a 4%–12% NuPAGE Bis‐Tris precast gel (Thermo Fisher Scientific; #WG1401BX10). After transfer in nitrocellulose, the membrane was boiled in PBS for 10 min, blocked for 1 hr and incubated with the appropriate primary antibodies.

*Inhibitors of NGF‐receptors used*: 200 nM K252a (Abcam, Cambridge, UK; #ab120419), 1 µM TAT‐pep5 p75^NTR^ (Millipore, Temecula, CA; # 506181; Yamashita & Tohyama, 2003), were added 30 min before Aβ and NGF.

*The following primary antibodies were used*: anti‐Aβ 1–16 1:1000 (clone 6E10 #SIG‐39320); anti‐TrkA 1:1000 (Millipore; #06–574), anti‐pTrkA 1:1000 (Y794; Rajagopal, Chen, Lee, & Chao, 2004) kindly provided by M. V. Chao (New York University School of Medicine, New York, NY) anti‐Akt 1:1000 (Cell Signaling Technology, Danvers, MA; #C67E7), anti p‐Akt 1:1000 (Cell Signaling Technology; #130386), ant‐Erk 1:1000 (Promega, Fitchburg, WI; #V114A), anti‐pErk 1:1000 (Cell Signaling Technology; #4370S), anti‐c‐Jun 1:1000 (Cell Signaling Technology; #60A8), anti‐phospho‐c‐Jun 1:1000 (Cell Signaling Technology; #9261), anti‐p75 1:1000 (Millipore; AB1554), anti‐GAPDH 1:20000 (Fitzgerald, Acton, MA; #10R‐G109a), anti‐tubulin 1:20000 (Sigma‐Aldrich; #T5168). After incubation with the appropriate HRP‐conjugated secondary antibody (Santa Cruz, Dallas, TX; anti‐mouse #sc‐2005, anti‐rabbit #sc‐2004), membranes were developed using ECL‐enhanced chemiluminescence kit (Bio‐Rad, Hercules, CA). Densitometric analyses were performed using the NIH ImageJ 1.44p software.

### 2.4 Immunocytochemistry

*Immunofluorescence for NGF receptors*: Primary microglia were plated on coverslips in 24‐well plates coated with poly‐d‐lysine (1 × 10^5^ cells/well) in culture medium. Cells were fixed with 2% PFA, and blocked for 1 hr at room temperature. *Primary antibodies* (O.N. – 4°C): anti‐Iba1 1:500 (WAKO, Osaka, Japan; #019–19741) or anti‐Iba1 1:500 (Abcam; #Ab107159), anti‐TrkA 1:100 (MNAC13 from Cattaneo et al. [1999]), anti‐P75 1:500 (Millipore; AB1554).

*Immunofluorescence for Aβ uptake*: Primary microglia were plated on coverslips in 24‐well (1 × 10^5^ cells/well) in culture medium. They were treated with 1 μM soluble 555‐labeled Aβ (s555‐Aβ; Anaspec, Fremont, CA; #As‐60480) or AβOs (Meli et al., 2014) and 100 ng/ml NGF for 3 hr. After fixation and permeabilization, cells were blocked for 1 hr and stained with primary antibodies anti‐Iba1 1:500 (WAKO, Osaka, Japan; #019–19741;) and with anti‐Aβ oligomers A13 1:1000 (Meli, Visintin, Cannistraci, & Cattaneo, 2009), then incubated with mouse antibody anti‐epitope V5 (Sigma‐Aldrich; #V8137;1:5000). Appropriate secondary antibodies were used (1:500) (anti‐rabbit Alexa‐Fluor 555, anti‐mouse Alexa‐Fluor 488, Thermo Fisher Scientific; A‐21428;A‐21201).

### 2.5 Immunofluorescence (IF) on slice

*IF for NGF receptors/microglia/astrocytes detection*: Adult (P80–90) C57BL6J mice were sacrificed with a lethal dose of carbon dioxide and immediately underwent a perfusion procedure. Dry ice frozen brains were cut into 40 µm coronal sections with a cryostat microtome (Leica Microsystems, Wetzlar, Germany) at −20°C, including neocortex. Sections were with a mix of primary antibodies in PBS 0.3% Triton X‐100 (Applichem, BioChemica, Darmstadt, Germany) overnight at room temperature. Microglia was stained with either rabbit anti‐Iba1 1:800 (Wako, Osaka, Japan, 019–19741) or rat anti‐CD11b 1:300 (Serotec; Kidlington, UK, MCA711). Astrocytes were stained with rabbit Anti‐Glial Fibrillary Acidic Protein 1: 500 (Dako, Cytomation, Glostrup, Denmark, Z0334) or goat Anti‐Glial Fibrillary Acidic Protein 1:300 (Santa Cruz Biotechnology; sc‐6170). NGF receptors were identified by anti‐TrkA 1:300 (clone MNAC13; Cattaneo et al., 1999) and anti‐P75 1:300 (Promega Corporation, Madison, WI, G3231). Sections were incubated for 2 hrs at RT in a mix of the appropriate secondary antibodies—anti‐mouse/rabbit/goat/rat Alexa‐Fluor 488/555/649 conjugated (Thermo Fisher Scientific; A‐21428 diluted 1:500). DAPI was applied for 5 min in the second rinse.

### 2.6 Flow cytometry for phagocytosis analysis of beads, dextran or Aβ

*Sample preparation*: Primary microglia were plated in six‐well plates at a density of 5 × 10^2^ cells/well. The fluorescent material to be phagocytosed was placed in the culture medium 3 hr after treatment with NGF 100 ng/ml. Beads were first opsonized in 50% FBS and PBS for 1 hr RT (Polybead DyedRed 6 µm, Polyscience, Warrington, PA; Cat#15714), counted with the Burker chamber and given to cells at a concentration of roughly three beads/cell. Dextran was used at 2.5 mg/ml (Thermo Fisher Scientific; #D1841 RhodamineB 70,000 MW) while HiLyte Fluor 555 Aβ42 (Anaspec, Fremont, CA; #As‐60480) was used 1 µM. Cells were exposed to the material for 1 hr, then they were washed extensively with PBS, and fixed with 2% paraformaldehyde for 7 min. Cells were washed again with PBS and collected for analysis. *Data acquisition*: A Sorter S3 (BioRad) with a single 488 nm (100 mW) excitation laser was used. The gating strategy was decided on the FSC and SCC scatter plots, in order to gate out debris. Filters were based on the emission spectra of the fluorochromes: RhodamineB for dextran, DyeRed for beads, HiLyte Fluor 555 for Aβ42—580–650 nm (red channel). The total amount of beads, dextran or Aβ internalized by cells was determined by analyzing the population positive for the fluorescent marker conjugated with the material. The analysis was performed using the FlowJo software (FlowJo, LLC, Ashland, OR).

*List of concentrations and time of treatment for inhibitors and activators of macropinocytosis and phagocytosis used to determine the specific process of internalization activated by NGF administration*: IFNγ 10 ng/ml (R&D, Minneapolis MN, USA Cat. Number 485‐MI), Amiloride 50 µM (Sigma‐Aldrich; #A3085), CytochalasinD 10 µg/ml (Sigma‐Aldrich; #C827). These were added 20 hr before phagocytosis assay. Rho/Rac/cdc42 Activator I (Cytoskeleton, Denver, CO; cat. #CN04) was added after a 2 hr FBS starvation period and 1 hr and 30 min before the beginning of the experiment with beads and dextran. Phorbol 12‐myristate 13‐acetate (PMA) 100 nM (Sigma‐Aldrich; #P8139) was added 3 hr before the assay.

### 2.7 Microarray transcriptome analysis

Primary microglia were treated with 100 ng/ml NGF for 2, 8, or 24 hr. RNA isolation, amplification, and labeling was performed using an RNeasy mini kit according to manufacturer's protocol (Qiagen, ‎Venlo, The Netherlands‎). Total RNA was isolated from these cells using Trizol (Invitrogen, Carlsbad, CA) and DNAse treated by Qiagen columns. Quality and integrity of each sample was checked using the Agilent BioAnalyzer 2100 (Agilent RNA 6000 nano kit): samples with a RNA Integrity Number (RIN) index lower than 8.0 were discarded. All the experimental steps involving the labeling, hybridization, and washing of the samples were done following the standard one‐color microAgilent protocol. The gene expression profiling was performed using the Microarray Agilent Platform. 200 ng of RNA was labeled with Low Input Quick Amp Labeling Kit One‐Color (Agilent Technologies, Santa Clara, CA), purified and hybridized overnight onto the Agilent 8X60K whole mouse genome oligonucleotide microarrays (Grid ID 028005) according to the manufacturer's instructions for one‐color protocol. The Agilent DNA microarray scanner (model G2505C) was used for slide acquisition and spot analysis was performed with Feature Extraction software ver 10.7 (Agilent Technologies). Data filtering and analysis were performed using R‐Bioconductor and Microsoft Excel. All the features with the flag gIsWellAboveBG = 0 (too close to background) were filtered out and excluded from the following analysis. Filtered data were normalized by aligning samples to the 75th percentile. Differentially expressed genes were selected by a combination of fold change and moderated *t*‐test thresholds (R Limma test *p* value <.05; |Log2 fold‐change|>1.0). Principal Component Analysis, Multidimensional Scaling, Hierarchical Clustering of samples and volcano plots were computed using the open source RStudio (Boston, MA).

### 2.8 Live cell imaging

Primary microglia were plated (3 × 10^4^ cells) on Glass Bottom Microwell Dishes (35 mm), coated with poly‐d‐lysine, and left overnight to rest. Cells were treated with 100 ng/ml NGF for 24 hr. Cells were imaged for 1 hr through a 40× objective with a Leica SP2 confocal microscope (1 frame each 30 s). Cell dynamics was analyzed using a homemade Python script (number of cells imaged per experiment = 29). Parameters: *Morphing speed* measures how many times, during the acquisition, cells change their morphology. Two extremes were fixed as opposite morphological endpoints: roundish and polarized (with at least two ramification). We measured how many times cells shift between these two cell configurations. The parameter was used to classify the speed of changes in morphology. *Cell membrane changes* describes how cells change their Area (A) normalized on cell perimeter (p), in particular we measured ΔA/p between two consecutive frames (1 frame/min), giving us an intermediary to monitor membrane motility.

For the experiment of Aβ lysosome colocalization, microglial BV‐2 cells were plated overnight in RPMI containing 2% FBS on pre‐coated culture plates. Cells were incubated with 1 µg/ml Aβ‐488 and 100 nmol/l Lysotracker‐Red (Thermo Fisher Scientific; #L12492) and imaged using a Leica SP2 confocal microscope (Leica Microsystems, Wetzlar, Germany) for 1 hr with a 63×/1.4NA HCX PL APO objective. We used BV‐2 cells instead of primary cultures of microglia since live imaging requires long hours and it is too damaging for primary cultures.

### 2.9 Intracellular Aβ clearance and degradation

BV2 cells were incubated in culture medium with 1 µM soluble Aβ42 (Anaspec, Fremont, CA; #As‐64129) and 100 ng/ml of NGF for 3 hr. Cells were then either collected (the 3 hr time point) or the medium was changed after extensive washes with PBS to ensure the removal of Aβ42 in the supernatant. Cells were collected and lysed in ice‐cold RIPA buffer (SDS 1%) after either 5, 9, or 21 hr of washout, in order to allow the measurement of the phagocytosed Aβ which could be either digested (and detected in the cell extracts) or expelled (and detected in the supernatant) at each time point. After brief sonication, they were collected by centrifugation at 13,000 rpm at 4°C for 15 min. The supernatant at each time point was also collected. Aβ42 levels in the cell lysates were determined by immunoblotting with the anti‐Aβ antibody 6E10 (clone 6E10 #SIG‐39320; 1:1000, Covance, Princeton, NJ). The samples were resolved with 4%–15% bis‐tris SDS‐PAGE. Aβ levels were measured and normalized on the housekeeping GAPDH total protein levels. The Aβ supernatant levels were measured using ELISA Kit (Human Aβ42 Invitrogen KHB3441). Optical density was read at 450 nm on a Bio‐Rad plate reader. BV‐2 cells—as opposed to primary microglia—were used because of the high number of cells needed for this experiment (RRR rule)—each time point is indeed a parallel experiment.

### 2.10 Aβ phagocytosis in *ex vivo* hippocampal slices

*Cx3Cr1‐GFP* mice were deeply anesthetized (20% urethane solution, 0.1 ml/100 g body weight) via i.p. and decapitated to perform the immediate dissection of brain tissue. Horizontal slices containing the hippocampal area (200 μm thick) were obtained by a vibratome (Leica VT1200S). All of the above steps were performed in ice‐cold ACSF solution (artificial cerebrospinal fluid, in mM: NaCl, 119; KCl, 2.5; CaCl_2_, 2; MgSO_4_, 1.2; NaH_2_PO_4_, 1; NaHCO_3_, 26.2; glucose, 10) bubbled with 95% O_2_/5% CO_2_. Slices were stored in a recovery chamber containing oxygenated ACSF at room temperature, for at least 30 min prior to the addition of 100 nM s555‐Aβ with or without 100 ng/ml of NGF. After 3 hr, slices were fixed in 4% PFA for 18 hr at 4°C. Slices were put in 30% sucrose/PBS, then they were sectioned into 45 µm slices using a Leica microtome.

### 2.11 Electrophysiological recordings from neurons

Adult C57BL6 male mice were deeply anesthetized with isoflurane inhalation, decapitated, and brains removed and immersed in cold “cutting” solution (4°C) containing (in mM): 126 choline, 11 glucose, 26 NaHCO_3_, 2.5 KCl, 1.25 NaH_2_PO_4_, 10 MgSO_4_, 0.5 CaCl_2_ equilibrated with 95% O_2_ and 5% CO_2_. Coronal slices (300 µm) were cut with a vibratome (Leica) and then incubated in oxygenated artificial cerebrospinal fluid (ACSF) containing (in mM): 126 NaCl, 26 NaHCO_3_, 2.5 KCl, 1.25 NaH_2_PO_4_, 2 MgSO_4_, 2 CaCl_2_ and 10 glucose, pH 7.4; initially at 32°C for 1 hr, and subsequently at room temperature, before being transferred to the recording chamber and maintained at 32°C. Recordings were obtained from visually identified pyramidal neurons in layer 2/3, easily distinguished by the presence of an emerging apical dendrite. Experiments were performed in the whole‐cell configuration of the patch‐clamp technique. Electrodes (tip resistance = 3–4 MΩ) were filled with an intracellular solution containing (in mM): K‐gluconate 135, KCl 4, NaCl 2, HEPES 10, EGTA 4, MgATP 4 NaGTP 2; pH adjusted to 7.3 with KOH; 290 mOsm. Whole‐cell voltage‐clamp recordings (–70 mV holding potential) were obtained using a Muticlamp 700B (Axon CNS, Molecular Device). Action potential independent spontaneous excitatory postsynaptic currents (mEPSCs), recorded in the presence of tetrodotoxin (TTX) 1 μM and the GABAA receptor antagonist picrotoxin (100 μM), were filtered at 1 kHz, digitized at 10 kHz, and recorded on computer using Digidata1440A and pClamp10 software (Molecular Device). Series resistances were not compensated to maintain the highest possible signal‐to noise and were monitored throughout the experiment. Recordings were discarded if Rs changed 25% of its initial value. Spontaneous events were detected and analyzed with Clampfit 10.4 using amplitude and area thresholds set as a multiple (3–4X) of the SD of the noise. Each event was also visually inspected to prevent noise disturbance of the analysis. Each slice received only a single exposure to NGF (20 g).

### 2.12 Electrophysiological recordings from microglia cells

Acute cortical slices (250 μm) were obtained from CX3CR1+/GFP male mice (P18–P30) using the identical experimental procedures described in the above paragraph (recordings from neurons). After recovering for at least 1 hr at RT, each slice was transferred in the recording chamber under the microscope and perfused (2 ml/min) with warmed ACSF (32°C). Visually identified GFP‐expressing cortical microglial cells were patched in whole‐cell configuration. Micropipettes (5–6 MΩ) were filled with solution containing the following composition (in mM): KCl 140, EGTA 0.5, MgCl_2_ 2, HEPES 10, and Mg‐ATP 2 (pH 7.3 adjusted with KOH, osmolarity 290 mOsm; Sigma‐Aldrich). Voltage‐clamp recordings were performed using a Muticlamp 700B (Axon CNS, Molecular Device). Currents were filtered at 2 kHz, digitized (10 kHz) and collected using Clampex 10 (Molecular Devices); the analysis was performed offline using Clampfit 10 (Molecular Devices). Slicing procedure might activate microglial cells especially near the surface of the slice, therefore recordings were performed on deep cells. Cells were clamped to a holding potential of −20 mV. The current/voltage (I/V) relationship of each cell was determined applying voltage steps from −140 to +60 mV (DVm 20 mV) of 250 ms duration with interval of 5 s after whole‐cell configuration was achieved (HP = −20 mV between steps). Current values for each given voltage step were measured in the last two‐thirds to avoid contamination of capacitance artefacts. Resting membrane potential and membrane capacitance were measured at start of recording. NGF was applied in bath for 10 minutes. One to four cells per mice were recorded. At least four animals per group were used.

### 2.13 Neuron/microglia co‐cultures

At DIV (days *in vitro*) 17–19 for neurons, primary microglia were seeded onto cultured hippocampal neurons (1 × 10^5^ cells/well). The culture was maintained in Neurobasal‐A supplemented with 2% B27, 2 mM l‐glutamine and 10 μg/ml gentamicin and used after 24 hr for experiments. Co‐cultures were treated with soluble Aβ‐555 (100 nM), and 100 ng/ml NGF for 3 hr, fixed in 2% PFA and 5% sucrose for 10 min, washed in PBS and blocked for 1 hr at room temperature in BSA 1%. Incubation with primary antibody was performed at the following concentrations: anti‐PSD95 1:500 (Abcam; ab9909), anti‐actin 1:500 (Sigma‐Aldrich; A‐3853;), anti GluA1 1:100 (Millipore; #AB1504;).

### 2.14 Chemical LTP

*Cx3Cr1‐GFP* microglia (2 × 10^4^ cells/well) were added to DIV 17 cultured hippocampal neurons. After 48 hr, the cultures were treated with soluble Aβ‐555 (100 nM), with or without 100 ng/ml NGF for 3 hr. GI‐LTP was induced as reported in the literature (Ahmad et al., 2012). Briefly, cultures were incubated for 15 min at room temperature in standard ACSF (in mM: 125 NaCl, 2.5 KCl, 1 MgCl_2_, 2 CaCl_2_) with 0.02 mM Bicuculline and 0.001 mM TTX, then washed with Mg‐free ACSF and treated for 7 min with Mg‐free ACSF supplemented with 0.2 mM Glycine, 0.02 mM Bicuculline. After 7 min of stimulation, cultures were washed once in ACSF and left in culture medium for 1 hr and fixed in 2% PFA for 10 min.

### 2.15 Measurement of inflammatory markers

Simultaneous detection of multiple cytokines was obtained using the Mouse Inflammation Antibody Array (Raybiotech, Norcross, GA; Canada; AAM‐CYT‐6). Primary microglia from B6129 mice were plated in a 6‐well at the concentration of 6.5 × 10^5^ cells/well in culture medium. After 18 hr, cells were serum starved for 4 hr, and later treated with Aβ 1 µM or 100 ng/ml NGF or Aβ and NGF simultaneously. Cells were lysed in ice‐cold RIPA buffer (50 mM Tris/HCl, 150 mM NaCl, 1 mM EDTA, 1% Igepal, 0.5% Sodium Deoxycholate, 0.1% SDS, Protease Cocktail inhibitor) and sonicated briefly, and then collected by centrifugation at 13,000 rpm at 4°C for 15 min. Arrays were incubated with the appropriate blocking buffer for 2 hr. Eighty µg of protein extract were diluted in blocking buffer and incubated with the array overnight at 4°C. Then, arrays were washed accordingly and incubated for 3 hr at room temperature with the Biotinylated Antibody Cocktail solution. After washing, arrays were incubated with HRP‐streptavidin for 2 hr and detected using the Detection Buffer. Images were captured using the Chemidoc detection system (Bio‐Rad).

### 2.16 Image analysis

**Figure 1:**
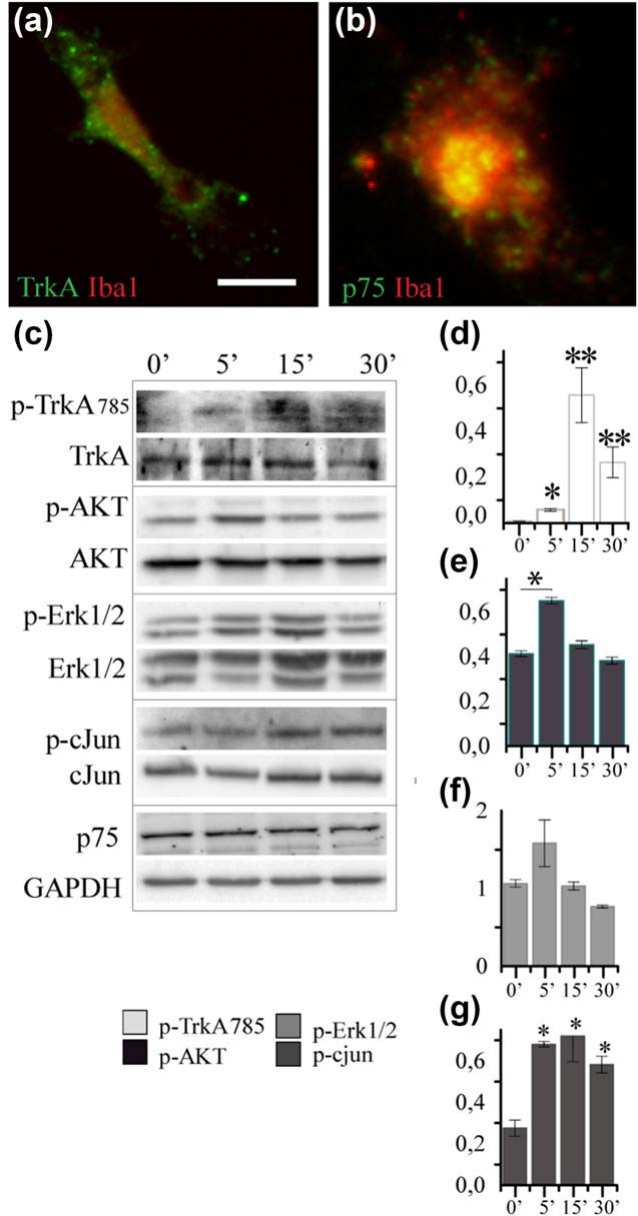
Primary microglia express NGF receptors and activate receptor‐mediated intracellular signaling after NGF stimulation. (a) TrkA and p75NTR (green) staining in primary microglia (red). (b) Western blots for proteins involved in NGF signaling transduction pathways: TrkA, AKT, Erk, and c‐jun. On the right, the histograms of the quantification of phosphorylated protein normalized on the total protein level (Data are mean ± *SD*; all data are representative of three independent experiments, *p < .05, **p < .01, Student's *t* test) [Color figure can be viewed at http://wileyonlinelibrary.com]

Experiments in Figures 1a and 9: 512 × 512 pixel images were acquired with a confocal microscope (Leica TCS SP2) using an oil objective: HCX PL APO 63.0× OIL (NA = 1.40), and pinhole was set to 1 AU. Sequential illumination with Ar 561 and Ar 488 laser lines was used to detect, sAβ‐555, ABOs, IBA1, TrkA, p75 immunofluorescence.

**Figure 2:**
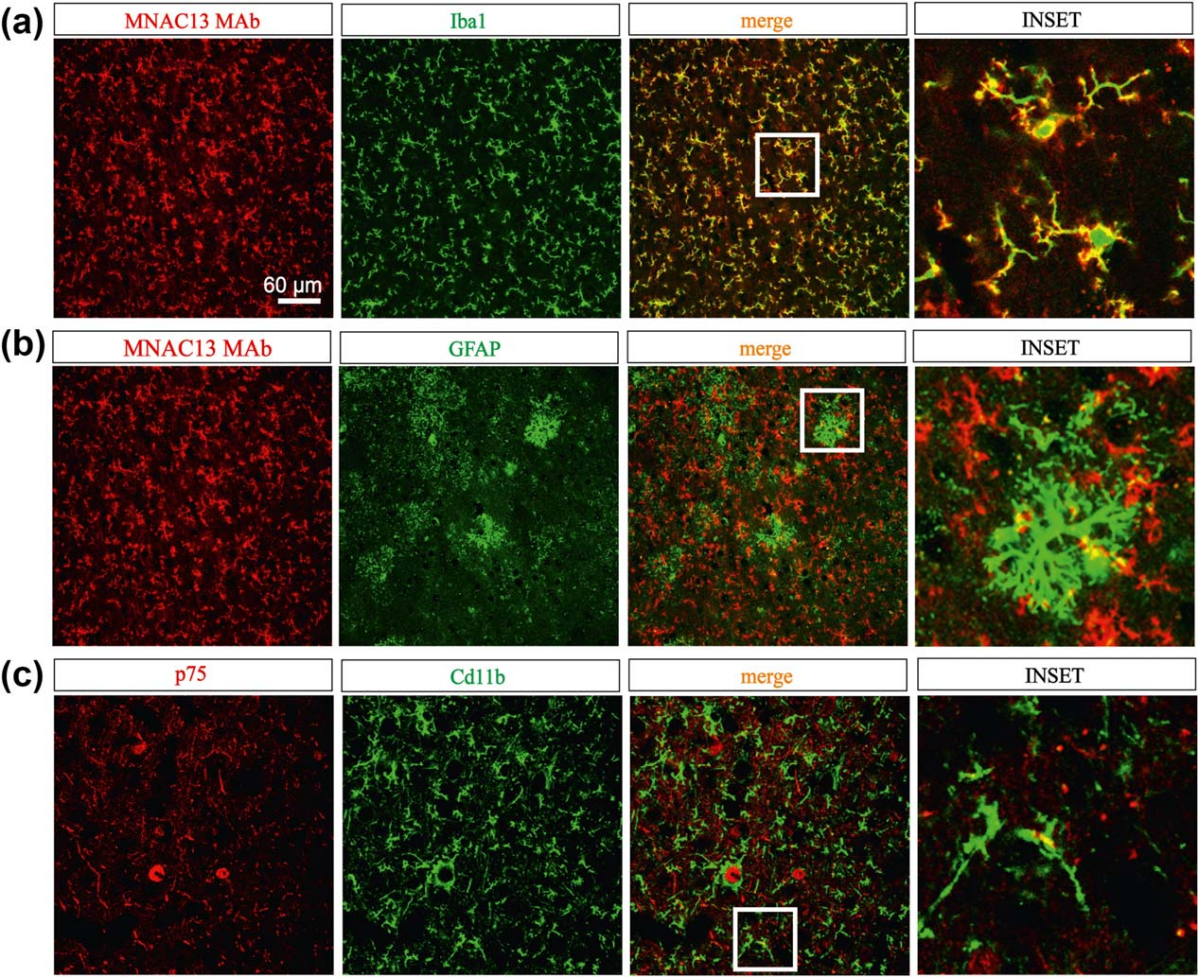
Expression pattern of NGF receptors in murine brain slices. (a) The anti‐TrkA MAb MNAC13 (red label) stains cortical microglia iba1 positive cells (green label). Merging areas (yellow label) and magnification (inset) show that the labeling involves both cytoplasm and fibers. (b) (middle panels) MNAC13 staining slightly overlapped with the astrocytic marker GFAP (in green). Below, (c) p‐75NTR (red label) is slightly expressed in CD11b^+^ microglia (green label) from cortical sections. Merging areas (yellow label) [Color figure can be viewed at http://wileyonlinelibrary.com]

Experiments in Figure 2: 2,048 × 2,048 pixel images were acquired with a confocal microscope (Leica SP5, Leica Microsystems, Wetzlar, Germany) equipped with four laser lines: violet diode emitting at 405 nm, argon emitting at 488 nm, and helium/neon emitting at 543 and 633 nm using a HCX PL APO 40× OIL objective, 1 zoom factor, pinhole 1 AU. Points of colocalization were supposed when a merging area in the same cell was evident, showing a yellow resulting color from the overlap of two green‐red signals, and they were verified by analysis on the *z*‐axes with 1 μm‐stacks.

Experiments in Figures 12, 13 and 14: 512x512 pixel images were acquired with a confocal microscope (Leica TCS SP5 on DM6000, equipped with MSD module) using an oil objective HCX PL APO CS 40.0× (NA = 1.25), digital and pinhole was set to 1.5 AU. Sequential illumination with HeNe 633, DPSS 561 and Ar 488 laser lines was used to detect Alexa647 (used for PSD95, actin and GluA1 immunofluorescence), sAβ‐555, and GFP or IBA1 immunofluorescence, respectively. The Aβ intracellular levels was quantified by measuring the mean 555 fluorescence intensity in the area circumscribed by microglial cell perimeter using the segmented line tool in ImageJ.

Dendritic spines were counted using ImageJ software. For this analysis, all dendritic protrusions with a clearly recognizable stalk were counted as spines. Spine number was divided by the length of the dendritic segment to generate dendritic spine density, expressed as number per micrometer. Chemical LTP was measured by quantifying the integral GluA1 fluorescence intensity of each spine.

### 2.17 Data analyses and statistics

Data are presented as means ± *SD* unless otherwise noted, using Origin (OriginLab Corporation, Northampton, MA). Means were compared using the unpaired or paired *t* test as indicated. Multiple comparisons were made using one‐way ANOVA test, followed by a *post‐hoc* Bonferroni test. The variance of each dataset was measured with an F test; *p < .05, **p < .01 and ***p < .001.

## 3 RESULTS

### 3.1 Microglia express NGF receptors *in vivo* and *in vitro*

The first step to validate microglia as NGF target cells was to ascertain whether they express functional NGF receptors. By immunocytochemistry, we could detect the expression of both TrkA and p75 in primary microglia (Figure 1a,b; see Section 2 for culture conditions). To assess the responsiveness of such receptors, primary microglia were treated with NGF and analyzed by WB (Figure 1c) at different time points. TrkA exhibited a significant time dependent activation upon treatment with the neurotrophin (measured as the ratio between phosphorylated TrkA and the total amount of TrkA; Figure 1d; *p < .05 at 5 min and **p < .01 at 15 and 30 min). Concerning the downstream intracellular signaling pathways activated by NGF in primary microglia, we could detect the activation of AKT and c‐jun signaling pathways (Figure 1e,f; *p* < .05), while the activation state of Erk remained unchanged (Figure 1g; *p* > .05).

We then proceeded to assess TrkA and p75NTR expression in adult *ex vivo* glial cells. In sections of mouse cortex, we detected colocalization of TrkA and Iba1 both in the cell bodies and on branches of microglial cells (Figure 2a; *n* = 3; three experimental replicates). In contrast, astrocytes (GFAP^+^ cells) showed a sparse overlapping with TrkA (Figure 2b; *n* = 3; three experimental replicates). Labeling of the p75 receptor showed some rare points of colocalization with CD11b^+^ cells (Figure 2c, *n* = 3/6) while no expression could be detected on astrocytes' bodies or branches (*n* = 3/3 data not shown).

Thus, we conclude that both *in vivo* and *in vitro* microglia possess NGF receptors, and—specifically in cell culture—we could observe standard receptor signaling in response to the neurotrophin, indicating that these receptors are indeed active.

### 3.2 NGF modulates the expression of genes involved in pathways of cell motility, phagocytosis and protein degradation

**Figure 3:**
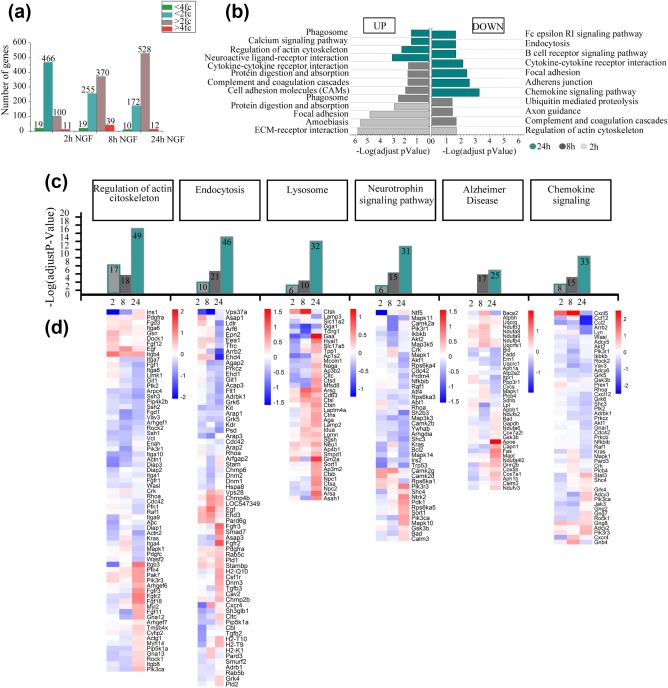
NGF modulates microglial gene expression. (a) The bar plot shows the global number of differentially expressed genes, up‐and down‐regulated by NGF at 2, 8, and 24 hr. Gene lists were selected using two different thresholds: 2.0 fold‐change in linear scale and Limma *p* value <.05 (blue for down‐regulated and plum for up‐regulated); 4.0 fold‐change in linear scale and Limma *p* value <.05 (green for down‐regulated and red for up‐regulated). (b) The horizontal bar plot shows the significantly enriched KEGG terms, following NGF treatment. Enriched pathways refer to up‐regulated genes (right bars) or down‐regulated ones (left bars), at 2, 8, and 24 hr (green, grey, light grey bars respectively). The analysis was performed on differentially expressed genes selected by two thresholds: corrected *p* value (FDR) <.05 and 1.0 fold‐change in linear scale. (c) The histograms show the adjusted *p* value (FDR) of selected enriched KEGG pathway at 2, 8, and 24 hr (green, gray, light gray bars respectively). Each bar contains the number of differential genes mapping to each specific pathway. Heatmaps show the Log2 fold‐change ratio of genes mapping to the corresponding modulated pathways on the top [Color figure can be viewed at http://wileyonlinelibrary.com]

To gain insight into potential functional microglial responses to NGF, gene expression profiling was performed on primary microglia treated with NGF (100 ng/ml) either for 2, 8, or 24 hrs (see Section 2). NGF induced global transcriptomic changes throughout the three time points. At two hours, the majority of differentially expressed genes (DEGs) were downregulated, while at 24 hrs there was a reversal, with a trend toward upregulation (Figure 3a). KEGG gene ontology analysis was performed, to cluster DEGs into pathways, thus identifying those primarily modulated by NGF.

At 2 hrs the majority of upregulated genes were linked to focal adhesion and extracellular matrix interactions, while downregulated genes were related to cytoskeletal rearrangements (Figure 3b). At 8 hrs, genes of *cell adhesion* molecules and of the *protein digestion* and *absorption* pathways were still upregulated (Figure 3b). At 24 hrs, the majority of upregulated genes were associated with the *regulation of actin cytoskeleton* and to the *phagosome* pathways, while downregulated genes belonged to *endocytosis*, *focal adhesion*, *adherens junction*, *cytokine‐cytokine receptor interaction*, and *chemokine signaling* pathways (Figure 3b).

We then focused on the analysis of specific gene clusters highlighted by the KEGG analysis—actin cytoskeleton regulation, endocytosis, chemokine signaling, protein digestion, neurotrophin signaling and genes linked to Alzheimer's disease (AD)—and represented the total amount of genes mapped to the specific KEGG category at each time point (Figure 3c) and the corresponding heat maps (Figure 3d). We found that, for each of these clusters, most changes occurred at 24 hrs. At this time, NGF induced a significant downregulation of rhoA and rock2, genes involved in actin dynamics (Julian & Olson, 2014; Sackmann, 2015), while most genes related to endocytosis and lysosomal activity, such as Gm2a (Sandhoff & Kolter, 1998), were up‐regulated. Concerning neurotrophins and AD pathways, we found an upregulation of sort1 and ApoE, respectively.

Finally, we looked at mRNAs involved in the inflammatory response, whose modulation is a major functional response of microglia. Interestingly, this mRNA class was not significantly represented among those upregulated by NGF. The largest modulation was actually the downregulated expression of cxcl5, ccl12, ccl2.

Overall, our data suggest that NGF might influence the motility, the phagocytic and protein degradation abilities of microglia, without activating them in the classical proinflammatory sense.

### 3.3 NGF enhances microglial membrane dynamics, but not their cell speed

The surveillance activity of microglia can be mediated either by the translocation of their cell body toward sites of injury, where chemoattractant substances are released, or by finer movements of their branches—by extension and retraction—in response to either physiological or pathological stimuli (Nolte, Möller, Walter, & Kettenmann, 1996; Stence, Waite, & Dailey, 2001). Since transcriptome analysis revealed changes in cytoskeletal related genes, we asked whether NGF might induce changes in cell body migration and/or in the motility of cell membrane and processes.

**Figure 4:**
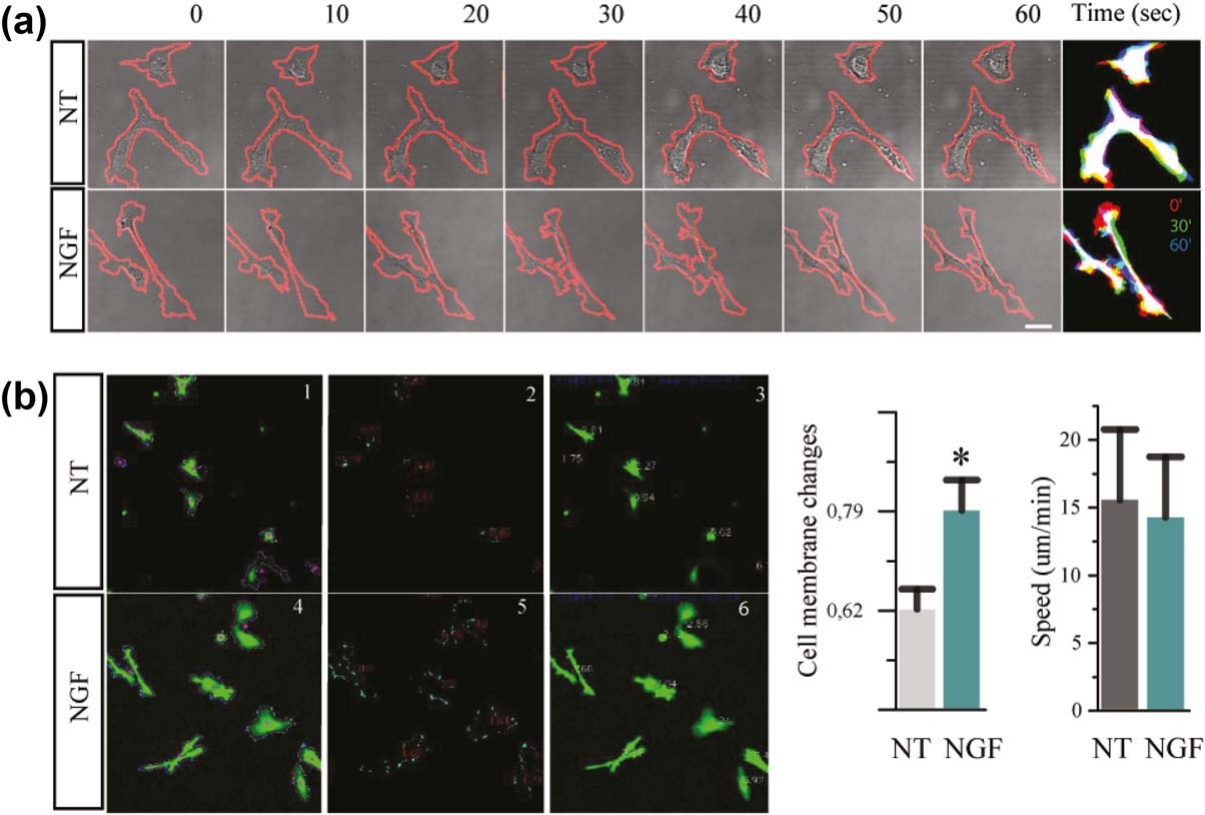
NGF modulates microglial motility dynamics. (a) Bright‐field image during a time lapse of primary microglia from CX3CR1‐GFP mice. (b) In the panel, it is shown the pattern recognition from a *Python based script* that describes (1–3) naive cells and (4–6) NGF cells. The boxes show in (1, 4) perimeter in violet and cell center in pink, (2, 5) perimeter difference between two consecutive frame, (3, 6) cell speed. The histograms show the plotted results of these parameters: cell membrane changes and speed (*n* = 29; data are mean ± *SD*; all data are representative of three independent experiments *p < .05, Student's *t* test) [Color figure can be viewed at http://wileyonlinelibrary.com]

The chemotactic properties of NGF specifically on rat microglial cells have been previously documented (De Simone et al., 2007). Our *in vitro* approach to assess the effects of NGF on motility—inspired from transcriptomics results—was that of operating *time‐lapse* recordings of NGF‐treated—freely moving – microglia (See Section 2 for culture conditions). Primary microglia were monitored for 1h in a culture chamber after treatment. Videos were analyzed by means of a Python script capable of extracting and quantifying useful features of the microglial motility behavior. This analysis unveiled that the speed of the cell body of NGF‐treated microglial cells was not significantly different from that of untreated microglia (Figure 4b), meaning there was no overall translocation from one place to another.

We thus concentrated on another parameter of cell motility: *cell membrane changes*. These structural changes occurred on a timescale of minutes and were evaluated as the difference in cell area between two consecutive frames (ΔA) normalized over the cell perimeter (p) (ΔA/p). Since this parameter evaluates the rate of change in cell area—a measure of its ability to elongate and retract—this can be thought of as an *in vitro* measure of exploratory behavior. We found that NGF‐treatment induced a significant increase in exploratory tendency in microglia (Figure 4b).

### 3.4 NGF promotes microglial macropinocytosis but not phagocytosis

**Figure 5:**
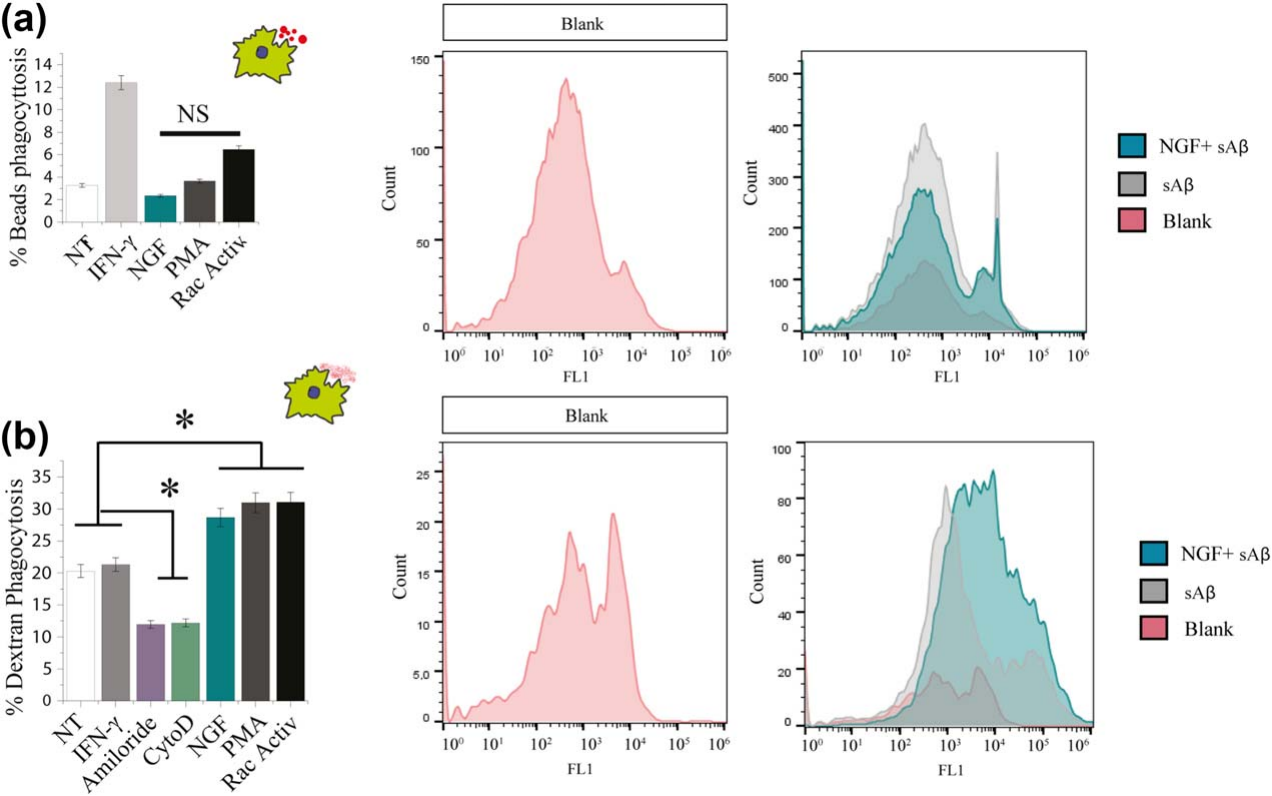
NGF enhances macropinocytosis of dextran but not phagocytosis of beads. (a) *Phagocytosis of beads*: Primary microglia from *CX3CR1‐GFP* mice were incubated with 6 µm beads and 10 ng/ml IFNγ, 100 ng/ml NGF, 100 nM PMA, and 1 µg/ml Rho/Rac/Cdc42 activator I for 3 hr. (b) *Macropinocytosis of dextran*: Primary microglia from *CX3CR1‐GFP* mice were incubated with 2.5 mg/ml Dextran and 10 ng/ml IFNγ, 50 µM Amiloride, 5 µg/ml Cytochalasin‐D, 100 ng/ml NGF, 100 nM PMA, and 1 µg/ml Rho/Rac/Cdc42 activator I for 3 hr. (mean ± *SD*, *p < .05, Student's *t* test) [Color figure can be viewed at http://wileyonlinelibrary.com]

Microglia are capable of engulfing material through three different mechanisms: phagocytosis, receptor‐mediated endocytosis and pinocytosis. Phagocytosis is used to internalize large particles (Stuart, Ezekowitz, & Alan, 2005), while pinocytosis is typically associated with the uptake of soluble substances, such as, for instance, soluble Aβ peptide (Mandrekar et al., 2009). We evaluated whether the observed NGF‐dependent changes in membrane motility might underlie changes in engulfing processes. To investigate this, we used an in vitro assay—followed by FACS analysis—where primary microglia were incubated with either fluorescent opsonized latex beads or dextran, in the presence or absence of NGF (see Section 2 for culture conditions). Beads are ingested through a phagocytosis process, while dextran through macropinocytosis (BoseDasgupta & Pieters, 2014), different engulfment processes that can be distinguished by means of known inhibitors and activators of the cellular mechanisms subserving them. IFN‐γ was used as a positive activation control for phagocytosis (Smith, van der Maesen, & Paul Somera, 1998), while PMA and Rac‐cdc42 activator‐I were used as positive activation controls of dextran macropinocytosis (BoseDasgupta & Pieters, 2014; Swanson, 1989). NGF was found not to increase the number of latex beads internalized by microglia (Figure 5a) but to selectively upregulate the internalization of dextran (Figure 5b). Thus, we conclude that NGF positively affects macropinocytosis though sparing other phagocytosis processes in primary microglia.

### 3.5 NGF activates microglia currents and modulates glutamatergic neurotransmission by acting on microglial cells

**Figure 6:**
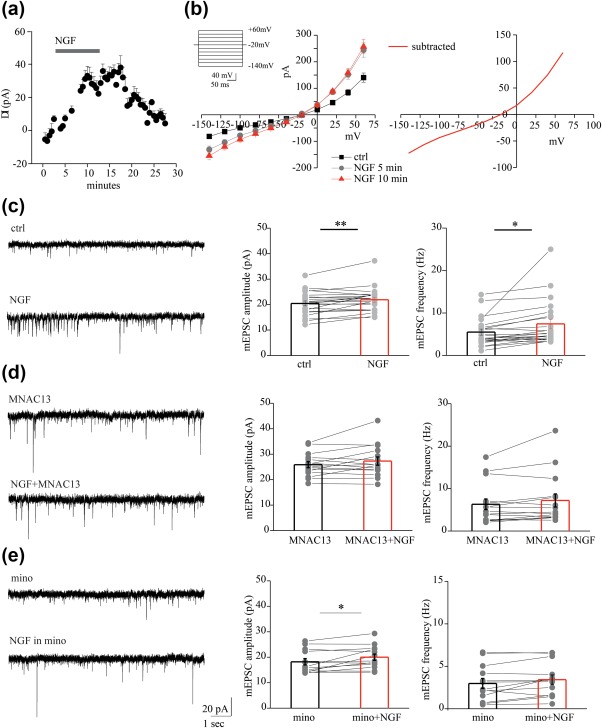
NGF affects microglial currents thereby enhancing excitatory neurotransmission. (a) Time plot of the mean current amplitude induced by NGF application recorded from microglial cells (*n* = 17). (b) Left, current‐voltage relationship of the NGF‐induced current by application of NGF (20 γ) in a microglial cell from acute cortical slice of CX3CR1^+^/GFP mouse before (black curve) and after 5 (light gray curve) and 10 min (red curve) NGF application. Right, NGF induces an outward rectifying current with reversal potential at about 15 mV at a holding potential = −20 mV (results obtained by subtracting the current before and after the NGF application). (c) Left, example traces of AMPAR mEPSCs recorded from a pyramidal neuron at −70 mV, in control (ctrl) and after NGF (20 γ), in the presence of picrotoxin (100 mM) and TTX (1 µM). Right, bar histograms of group data showing the NGF‐mediated increase of mEPSCs amplitude and frequency. (*n* = 22, *p < .05, **p < .01, paired sample *t* test. (d) Same as in c but in the presence of the anti‐TrkA, MNAC13. Note that 20 γ NGF did not enhance mEPSC amplitude and frequency when TrkA receptors are blocked (*n* = 15, *p* = .012 and *p* = .7 for amplitude and frequency, respectively; paired sample *t* test). (e) Left, example recordings of mEPSCs before and during NGF in the presence of 100 nM minocycline (mino). Right, population plots of mEPSC amplitude and frequency in minocycline, before (black bar) and during NGF (red bar), showing that NGF increased selectively the mEPSC frequency but not the amplitude when microglia activation was blocked (*n* = 13, *p* < .05 and *p* = .5, paired sample *t* test. Data are values from single cells (gray filled circle) and mean ± *SEM* (bars) [Color figure can be viewed at http://wileyonlinelibrary.com]

An *ex vivo* correlate to microglial behavior in response to NGF was obtained by performing patch clamp recordings from microglia in acute brain slices. Our data reveal that NGF triggers an outward current (Figure 6a). To study changes in this outward NGF‐induced current, we repetitively clamped the membrane from a holding potential of −20 mV to a series of hyperpolarizing and depolarizing voltage steps before and after the application of NGF (Figure 6b, left inset). The current‐voltage clamp curve of the response to NGF was outward slightly rectifying and reversed at 15 mV (*n* = 17, *p* < .05, Figure 6b). At a holding potential of −70 mV, NGF induced a current that reverses at 25 mV (*n* = 17, data not shown). These data reveal that NGF modulates microglial currents and as such can be considered functionally active on microglia in an *ex vivo* setting.

Emerging evidence is showing that stimulation of microglia by activation of glial receptors affects neurotransmission (Marrone et al., 2017; Riazi et al., 2015). Therefore, we hypothesized that also NGF may indirectly modulate glutamatergic neurotransmission by acting on microglial cells. To test this possibility, we first investigated the action of NGF on miniature excitatory postsynaptic currents (mEPSCs) recorded from pyramidal neurons. Bath application of NGF (2 μg/μl) for ten minutes significantly increased both the amplitude and frequency of mEPSCs (from 20.45 ± 0.97 to 22.90 ± 1.00 pA and from 5.50 ± 0.71 to 7.43 ± 1.14, *n* = 22; *p* < .01 and *p* < .05 respectively; Figure 6c). These enhancements were, at least partly, due to TrkA receptor activation, since anti‐TrkA mAb MNAC13 counteracted the increase of both amplitude and frequency by NGF (from 25.85 ± 1.188053 to 26.28981 ± 1.621783) and frequency (from 6.30 ± 1.249805 to 7.10 ± 1.535248, *n* = 15; *p* = .12 and *p* = .07 respectively; Figure 6d). Then, we carried out experiments in the presence of minocycline, which prevents microglia activation (Plane, Shen, Pleasure, & Deng, 2010). Minocycline (100 nM) inhibited the NGF‐induced increase of mEPSC frequency, without affecting the rise in amplitude (from 2.98 ± 0.58 to 3.43 ± 0.55 Hz and from 18.21 ± 1.25 to 20.01 ± 1.23, *p* = .5 and *p* < .05 respectively). Altogether, these data strongly suggest that NGF acts on microglia to modulate glutamatergic neurotransmission.

### 3.6 NGF and microglia in pathological conditions: Alzheimer's disease

Having established that NGF modulates microglial activity in physiological conditions, we then assessed the effect of NGF on microglia in a pathology‐related context, such as Alzheimer's disease. Microglia are important players in the pathogenesis of neurodegenerative disorders and they are being studied either as promoters of disease or physiological tools to be exploited to help with disease outcome.

### 3.7 NGF counteracts Aβ proinflammatory effect on microglia

**Figure 7:**
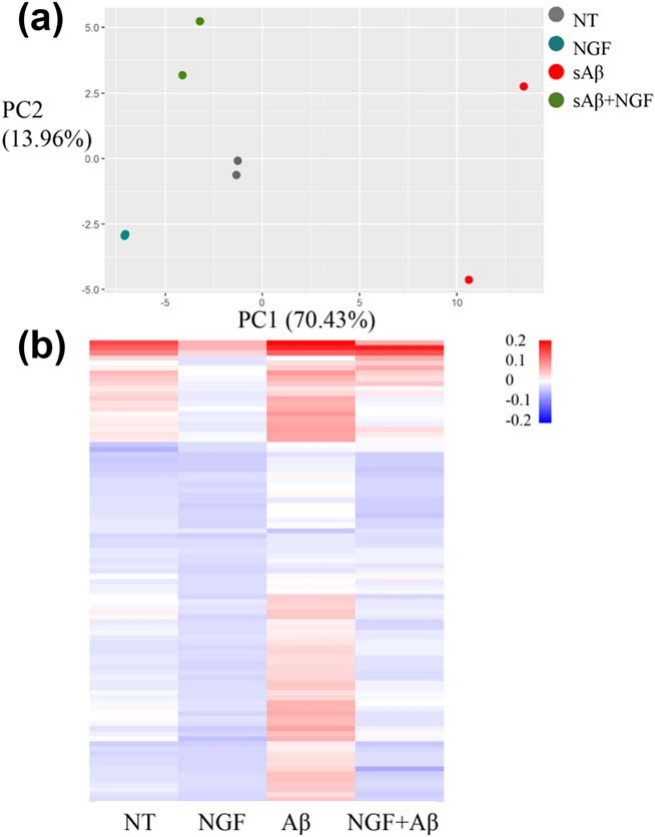
Anti‐inflammatory effect of NGF on microglia primed with Aβ. (a) PCA analysis of the inflammatory array. In the graph, two biological replicates of four different treatments were plotted. PC1 and PC2 represent the first two principal components, the proportion of variance (POV) held by these components is reported in brackets as percentages. (b) Inflammation array of primary microglia treated with NGF, Aβ, or Aβ and NGF reported as heatmaps, the scale bar represents the minimum and maximum levels of protein mean. Analysis was performed on RStudio (Boston, MA) [Color figure can be viewed at http://wileyonlinelibrary.com]

The amyloid‐β peptide provides an inflammatory stimulus to microglia (Combs, Karlo, Kao, & Landreth, 2001). Given the above‐mentioned effects of NGF on microglial cells, it was of interest to ask whether and how NGF can modulate their Aβ‐induced inflammatory profile. To this aim, we investigated the expression of inflammatory cytokines and chemokines in primary microglia in response to NGF, Aβ and Aβ with NGF, with an inflammation antibody array (See Section 2 for culture conditions). Looking at the heatmaps (Figure 7b), we can macroscopically see the pro‐inflammatory activity of Aβ by the prevalence of the red bars (increased quantity of cytokines). It is apparent that NGF carries out the opposite effect: not only it is intrinsically anti‐inflammatory when administered on his own but, given in concomitance with Aβ, NGF treatment effectively counteracts Aβ‐induced pro‐inflammation, returning cytokines to levels of untreated microglia. This effect was quantified by the PCA analysis (Figure 7a), that shows NGF treated cells to be at opposite sides of the PC1/PC2 plane, with untreated cells having an intermediate position—closer to the NGF groups—and Aβ treated cells clustering elsewhere (the list of values with each specific cytokine is provided as Supporting Information).

Thus, NGF is very effective in reverting the microglial pro‐inflammatory state induced by Aβ, while it has a moderate effect on the inflammatory phenotype of naive cells, consistent with the transcriptomic study we presented in Figure 3.

### 3.8 NGF promotes the internalization of soluble Aβ oligomers through TrkA signaling

**Figure 8:**
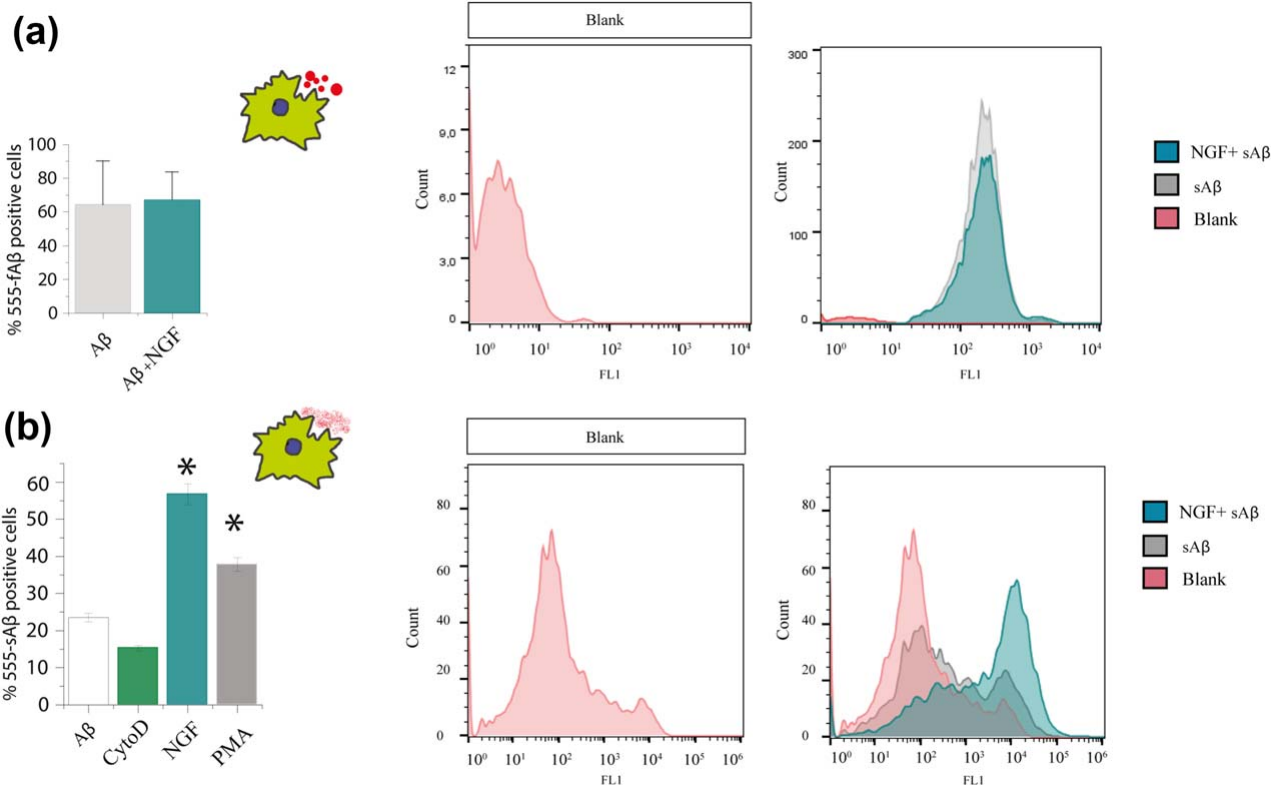
NGF increases the macropinocytosis of soluble Aβ but not the phagocytosis of fibrillar Aβ. Primary microglia from wild type mice were incubated with 1 µM of fAβ or sAβ and 100 ng/ml NGF. Uptake was quantified using flow cytometry and compared with control non treated cells (a) Internalization of fAβ is not affected by NGF treatment. (b) Internalization of the soluble peptide is increased after NGF treatment. *Controls*: NGF has a similar effect to PMA (an activator of macropinocytosis), CytochalasinD: inhibitor of endocytic processes (mean ± *SD*, *p < .05, Student's *t* test) [Color figure can be viewed at http://wileyonlinelibrary.com]

Microglia play an important role in the engulfment of different forms of the Alzheimer's hallmark Aβ peptide. While microglial cells endocytose fibrillar Aβ by phagocytosis, the soluble forms of the Aβ peptide are engulfed by macropinocytosis (Mandrekar et al., 2009). Thus, we asked whether NGF, which our previous experiments have shown to increase macropinocytosis, differentially regulates the engulfment of fibrillar (fAβ) and soluble Aβ (sAβ). To this aim, we incubated primary microglia with either fluorescent fAβ or sAβ and we tested the effect of NGF by FACS, IF and WB (see Section 2 for culture conditions). Consistently with our previous results (Figure 5), FACS analysis revealed that NGF did not increase the engulfment of fAβ (Figure 8a) but increased significantly the macropinocytosis of sAβ (Figure 8b).

**Figure 9:**
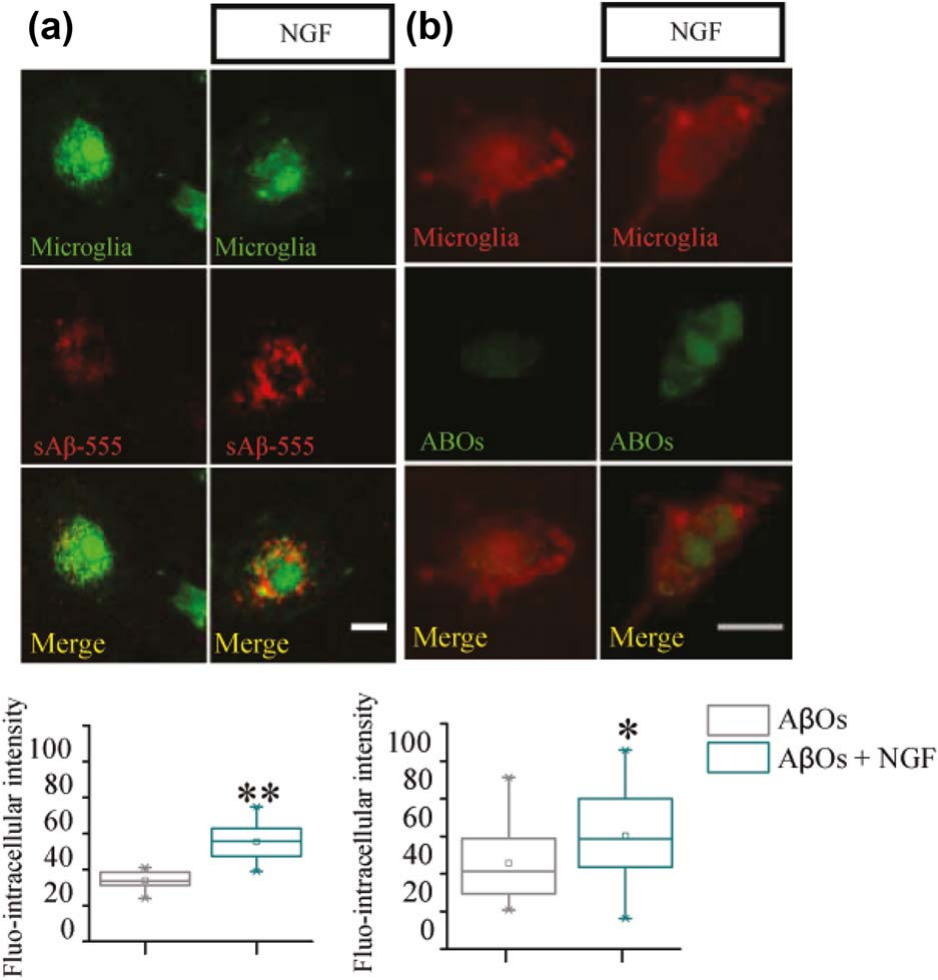
NGF increases the engulfment of sAβ peptide and ABOs: Immunofluorescence. Primary microglia from *CX3CR1‐GFP* (a) and wild‐type mice (b) were incubated respectively with 1 µM of fluo‐555 sAβ peptide and ABOs, from 7pA2 supernatant, in presence or absence of 100 ng/ml NGF (10 µm scale bar, 20 < *n* < 30, *p < .05 **p < .001, Kolmogorov‐Smirnov test) [Color figure can be viewed at http://wileyonlinelibrary.com]

The levels of sAβ and AβO—pure oligomers produced in vitro (Walsh et al., 2002)—inside primary microglial cells (from both B6129 and Cx3Cr1‐GFP mice) was also measured by immunofluorescence with anti‐oligomer scFv A13 (Meli et al., 2009) confirming an increase in the internalization of the soluble peptide after NGF treatment (Figure 9a,b).

**Figure 10:**
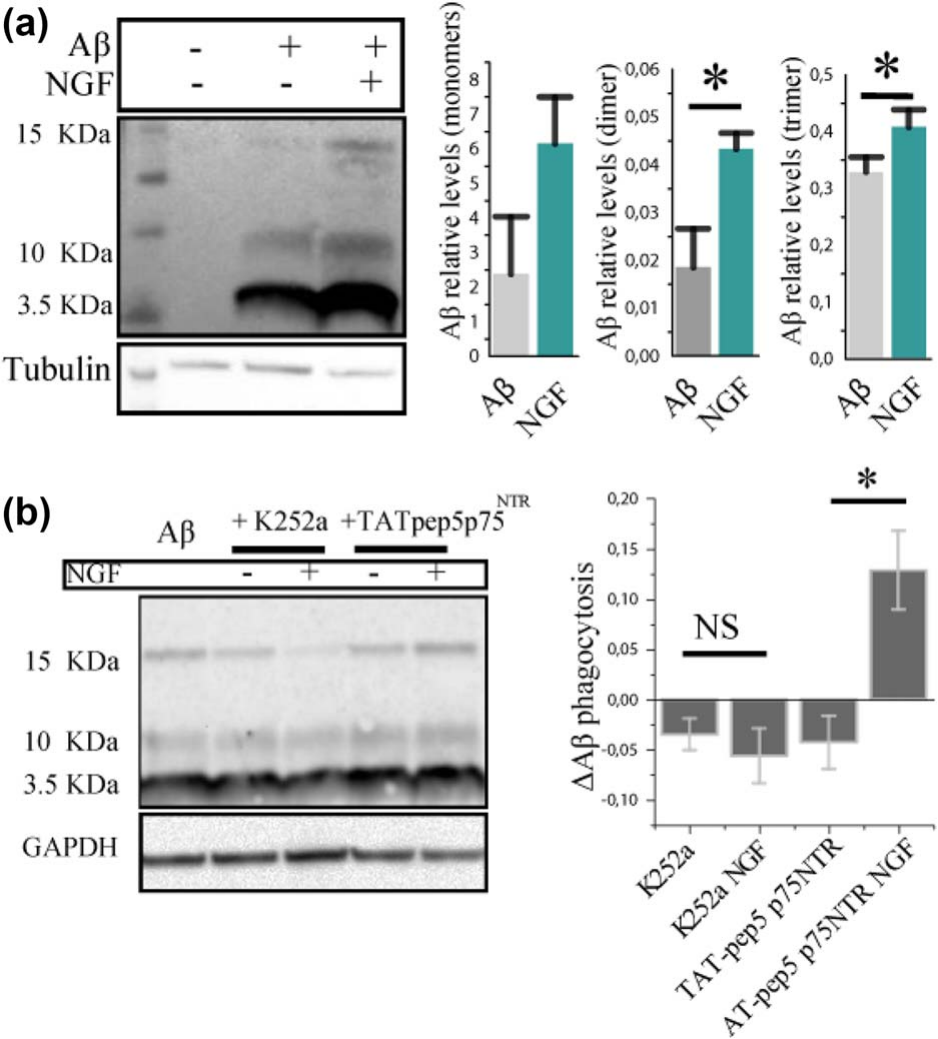
The modulation of microglial phagocytic activity is TrkA dependent. (a) Western blot of primary microglia treated with 1 µM of sAβ with or without NGF. Values are expressed as relative levels to controls (mean ± *SD*, *p < .05, Student's *t* test). (b) Primary microglia treated with 200 nM K252a, intracellular TrkA inhibitor and with 1 µM TAT‐pep5 p75^NTR^, inhibitor of p75NTR intracellular signaling. Values are normalized to the signal of samples treated with only sAβ (mean ± *SD*, *p < .05, Student's *t* test) [Color figure can be viewed at http://wileyonlinelibrary.com]

To distinguish different Aβ species, we performed western blot analysis for Aβ on cell extracts. We found that NGF determines a two‐fold increase of the internalized Aβ dimers and trimers (Figure 10a). To discern the involvement of the different NGF receptors in the internalization of Aβ, we interfered with TrkA and p75NTR signaling through specific inhibitors: K252a, which blocks TrkA phosphorylation and signaling, and TAT‐pep5, a p75NTR signaling inhibitor. K252a, and not TAT‐pep5, was able to block the increase in the uptake of AβOs in response to NGF (Figure 10b). Thus, we conclude that NGF is able to increase selectively macropinocytosis of soluble Aβ oligomers in primary microglia by a TrkA‐dependent mechanism.

### 3.9 The fate of internalized sAβ following NGF treatment

What are the consequences of the increased macropinocytosis of Aβ oligomers induced by NGF? The Aβ engulfed could be either accumulated inside the cells, expelled through exocytosis/released in exosomes, or digested. Transcriptome analysis revealed a strong modulation by NGF of genes involved in protein digestion, giving us cause to test the hypothesis that, in addition to *s*Aβ internalization, also the degradation of internalized *s*Aβ might be modulated in response to NGF.

**Figure 11:**
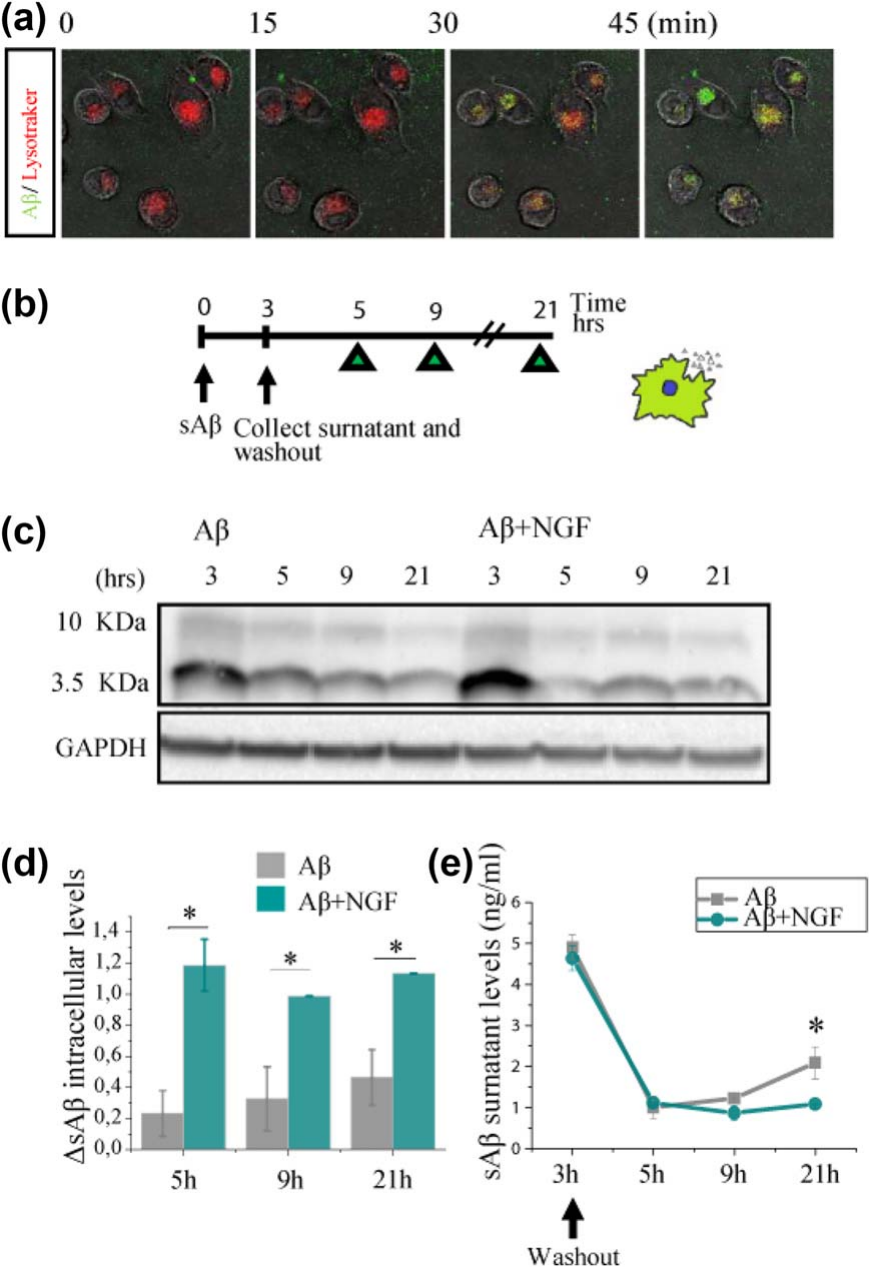
*s*Aβ is digested and not released in the extracellular environment in cells treated with NGF in BV2 microglial cells. (a) Soluble Aβ is rapidly trafficked to lysosomes for degradation. Confocal imaging of live BV‐2 microglia 45 min of 1 µM soluble Aβ1–42‐488 demonstrated localization of Aβ (green) within lysosomes. Lysosomes were stained using LysoTracker (red). (b) Experimental design for the degradation experiment (c) Western blot of cells lysate. (d) The histogram shows the degradation measure as the delta between the protein levels at the *n* time point and *(n +* 1*)* time point and thus represents the amount of protein that has been digested from one time point to the other. (e) Extracellular Aβ levels measured by ELISA. The data represent the outcome of three independent experiments (mean ± *SD*, *p < .05) [Color figure can be viewed at http://wileyonlinelibrary.com]

We followed the fate of sAβ using lysotracker, a dye that marks lysosomes. We used, for this experiment, microglia BV2 cells, that also display functional TrkA and p75 receptors (Supporting Information). The *s*Aβ peptides (green) internalized by BV2 microglial cells following NGF incubation is increased with respect to control. Moreover, the internalized Aβ colocalizes with lysotracker (red), suggesting that the engulfed material might go through lysosomal degradation (Figure 11a). In order to quantify such degradation and the hypothetical release of Aβ—such as suggested by (Joshi et al., 2014)—we proceeded as follows (Figure 11b): BV2 microglial cells were treated with soluble Aβ for 3 hr, then supernatant was collected and cells were washed to remove the Aβ excess (see Section 2 for culture conditions). We then monitored Aβ intracellular and extracellular levels in parallel experiments at 5, 9, and 21 hr, by WB of cell extracts—reflective of degradation—and ELISA of supernatants—to detect material that was expelled.

This experiment reveals that not only NGF‐treated cells ingest more Aβ than non‐treated cells (Figure 11c,d)—as expected—but also that NGF‐treated BV2 microglial cells digest a greater amount of Aβ (Figure 11d) and release a smaller fraction of it into the extracellular compartment, compared with untreated control cells (Figure 11e).

### 3.10 NGF increases internalization of sAβ by microglia in *ex vivo* brain slices

**Figure 12:**
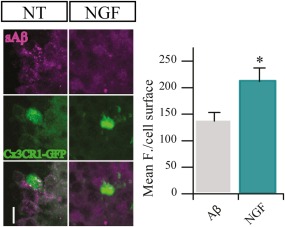
NGF increases the engulfment of sAβ *ex vivo*. Representative images of confocal stack acquisitions from 45 µm Cx3Cr1‐GFP slices. *Ex vivo* 200 µm slices were first treated with 0.1 µM sAβ and with/without of 100 μg/ml NGF then analyzed by IF for Aβ content (mean ± *SD*, *p < .05, Student's *t* test) [Color figure can be viewed at http://wileyonlinelibrary.com]

A big question was then to assess whether NGF effect on Aβ internalization might also be active on microglial cells integrated in the physiological circuit of the brain. To this aim, Aβ phagocytosis was tested in an *ex vivo* setting, in acute brain slice preparations from CX3CR1‐GFP adult mice. The acute slice was incubated for 3 hrs with NGF and fluorescent s555‐Aβ, the slice was then fixed, cut to 45 μm thick slices and mounted on glass slides to quantify internalization of fluorescent Aβ by GFP^+^ cells. We found a significant increase of internalized Aβ in microglial cells from brain slices that were incubated with NGF (Figure 12), demonstrating that, indeed, the modulatory effect by NGF can translate to microglia *in vivo*.

### 3.11 NGF protects against Aβ‐induced spine toxicity and rescues spine density and LTP deficit, in a microglia‐dependent way

**Figure 13:**
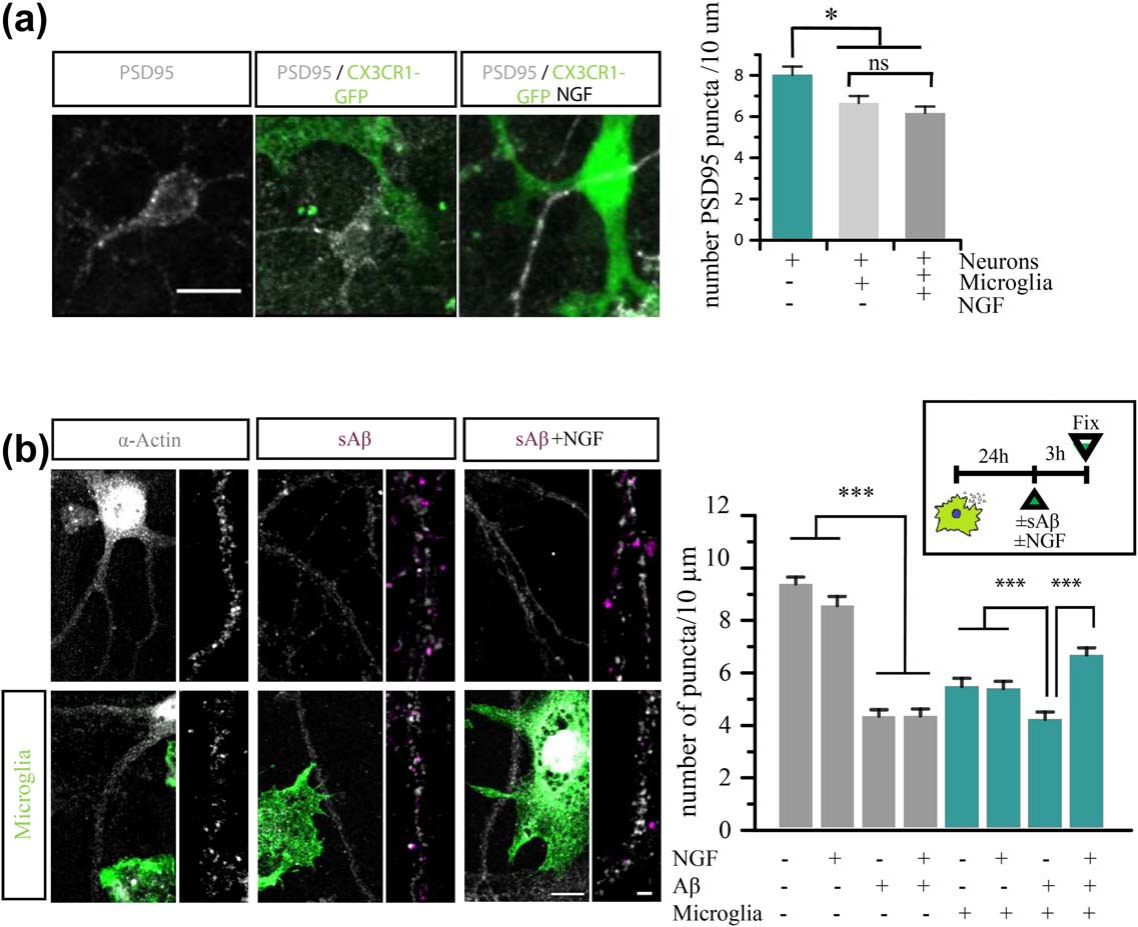
NGF protects against Aβ‐induced spines toxicity in a microglial dependent fashion. (a) Representative images from confocal acquisition show PSD95 (white) puncta in neurons and in co‐cultures +/− NGF. (b) Representative images of confocal acquisition of neuronal spines labeled with actin (white) and the dendrites magnification +/− sAβ (violet) in microglia (green)‐neuronal co‐cultures; (200 < *n* spines <500 for two independent experiments, mean ± *SED* *p < .05 ***p < .001, one‐way ANOVA test, followed by a *post‐hoc* Bonferroni test) [Color figure can be viewed at http://wileyonlinelibrary.com]

It is known that Aβ oligomers decrease spine density both in vitro and in vivo, and impair synaptic long‐term potentiation (LTP; Jekabsone, Mander, Tickler, Sharpe, & Brown, 2006; Palop & Mucke, 2010; Selkoe, 2008; Wei et al., 2010). Moreover, in the healthy developing and adult brain, an established physiological function of microglia is precisely the regulation of synapse number—*synaptic pruning* (Paolicelli et al., 2011; Parkhurst et al., 2013; Sipe et al., 2016; Zhan et al., 2014). Therefore, we asked whether NGF might regulate the activity of microglia on spines. To this aim, we performed co‐cultures of primary microglia with mature neurons and we quantified spine density following NGF treatment (Figure 12a; see Section 2 for culture conditions). After 24 hrs of co‐culture, the number of PSD95 positive puncta was lower on neurons cultured with microglia than in control neuronal cultures; this reflects the normal phagocytic activity of microglia on synapses (Ji, Akgul, Wollmuth, & Tsirka, 2013). In contrast, NGF treatment of microglia does not determine any further reduction—nor increase—of spine number compared with untreated microglia‐neuron co‐cultures (Figure 13b). Thus, NGF does not modulate the phagocytosis of synapses by microglia.

We next asked if NGF‐treated microglia could rescue spine loss mediated by sAβ exposure. Surely, in our control experiment in pure neuronal cultures, sAβ significantly decreases spine density by 50%, a decrease that could not be rescued by NGF treatment; however, in neuron‐microglia co‐cultures, where Aβ‐induced spine loss could still be detected, concomitant treatment with NGF completely prevented the decrease in spine density, demonstrating microglia as the mediator of NGF neuroprotective activity (Figure 13b). We conclude that NGF can prevent Aβ‐mediated spine loss in a microglia‐dependent manner.

**Figure 14:**
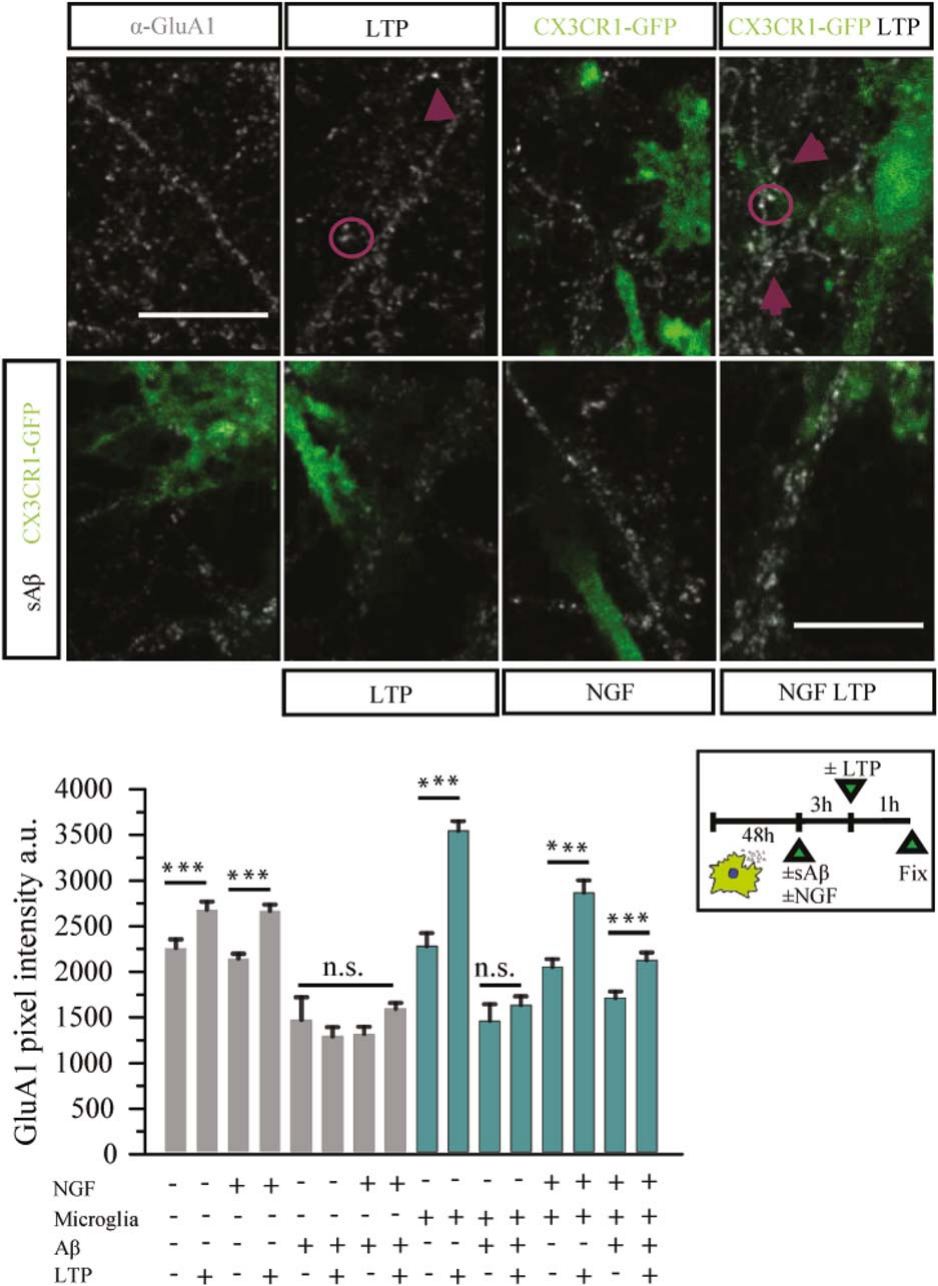
NGF protects against Aβ‐induced loss of potentiation in neuron‐microglia cocultures. (a) Effect of GI‐ LTP induction on GluA1 receptors (white) in different conditions, +/− microglia (green), +/− NGF, +/− sAβ. Histograms show the values of each experimental condition. (200 < *n* spines < 500 for two independent experiments, mean ± *SED* *p < .05 ***p < .001, one‐way ANOVA test, followed by a *post‐hoc* Bonferroni test) [Color figure can be viewed at http://wileyonlinelibrary.com]

The effect of Aβ on spine number is paralleled by its negative effects on synaptic potentiation in plasticity paradigms (Chen et al., 2000; Walsh et al., 2002). We therefore sought to investigate the interplay between NGF, microglia and spines in a plasticity protocol. We quantified synaptic potentiation measuring the total amount of GluA1 AMPA receptors in neurons under resting conditions or after glycine‐induced chemical LTP (GI‐LTP; as in Fortin et al., 2010; Ahmad et al., 2012). As previously reported, the staining intensity of synaptic GluA1 AMPA receptors increased in pure neuronal cultures 1 hr after GI‐LTP induction (Figure 14). Under our conditions, in microglia‐containing cultures, neurons were found to be more sensitive to GI‐LTP induction: GI‐LTP induced a greater increase of GluA1 synaptic staining (36% increase with respect non‐LTP cultures), when microglia were present, compared with control cultures without microglia (15.76% with respect to non‐LTP cultures; Figure 14). This suggests an enhancement of synaptic potentiation by microglia, an *in vitro* correlate of the evidence suggesting a role for microglia in spine formation and potentiation *in vivo* (Miyamoto et al., 2016; Parkhurst et al., 2013). sAβ exposure prevented the spine potentiation by GI‐LTP, since the levels of synaptic GluA1 were not significantly different between glycine‐stimulated and control cultures. The presence of microglia alone was not sufficient to rescue the synaptic GluA1 levels after sAβ incubation in sister cultures containing sAβ and microglia (Figure 14). Instead, NGF‐stimulated microglia cells were able to fully rescue the impairment of synaptic potentiation caused by sAβ. In fact, in NGF‐treated microglia‐neurons co‐cultures, synaptic GluA1 levels were significantly higher after GI‐LTP, even in the presence of sAβ (Figure 14). This was not due to a direct action of NGF alone on neurons, since in pure neuronal cultures NGF exposure was not sufficient, *per se*, to drive a significant change of synaptic GluA1 levels after GI‐LTP in the presence of sAβ (Figure 14). From these data, we conclude that—in our *in vitro* model of *neuroimmune* interface—not only NGF‐stimulated microglial cells are able to block spine loss induced by sAβ (Figure 13a,b), but they can also attenuate the sAβ‐mediated impairment of spine potentiation (Figure 14), most likely by sAβ removal from the neuron surrounding.

## 4 DISCUSSION

In this article, we have provided stringent evidence that microglia are target cells for NGF—both *in vitro* and *ex vivo*—and that the activity carried out by this neurotrophin might result neuroprotective and anti‐inflammatory in the context of Alzheimer's disease‐related insults. Indeed, we found that NGF is very effective in reverting the pro‐inflammatory state of microglia induced by Aβ, while it has only a moderate effect on the inflammatory phenotype of naive microglial cells.

This finding is highly relevant because, depending on their activation state and environment, microglia can either be beneficial or detrimental for brain physiology (Salter & Stevens, 2017). Indeed, recent genetic studies have underscored the emerging role of microglia in Alzheimer's disease pathogenesis (Keren‐Shaul et al., 2017). Microglia lose their amyloid‐β‐clearing capabilities with age and as AD progresses (Galatro et al., 2017; Krabbe et al., 2013). Therefore, affecting microglial homeostatic activities offers a potentially promising therapeutic avenue for AD pathology. However, approaches currently pursued to stimulate innate immunity via the Toll‐like receptor (TLR) pathway, such as the use of class B CpG (cytosine‐phosphate‐guanine) oligodeoxynucleotides (ODNs; Scholtzova et al., 2014, 2017, 2009), suffer from the problem that TLR ligands need to be very carefully titrated, to avoid excessive microglial stimulation. Indeed, while stimulation of innate immunity via TLR signaling pathways has been shown to be sometimes beneficial in modulating AD pathology (Richard, Filali, Prefontaine, & Rivest, 2008; Su, Bai, Zhou, & Zhang, 2016), it can also exert adverse effects in AD models (Campbell et al., 2009; Heikenwalder et al., 2004; Lee et al., 2008; Su et al., 2016).

In this respect, according to the results presented here, NGF appears to be able to stimulate an anti‐inflammatory response in microglia and to steer them to a fully neuroprotective phenotype, at many different levels, including cytokine and chemokine profile, motility, electrophysiological properties, engulfment of extracellular material, interactions with neurons and dendritic spines. Most notably, the inflammation‐modulating actions of NGF, such as for instance the transcriptomic changes, cytokine profile and the dendritic spine engulfment, are much more pronounced on Aβ treated microglia than on naive microglia. These properties of NGF could be exploited to harness the brain innate immunity as a safer *in loco* neuroprotective agent.

Our analysis shows that the receptor‐mediated signaling activated by NGF in microglia regulates a number of physiological activities of these cells, even triggering an outward rectifying membrane current. Although the identity of this current remains to be determined, the value of its reversal potential leads to propose that this NGF‐induced current may be subserved also by chloride channels (Murana et al., 2017).

Microglial activity is intimately associated with morphological changes (Nimmerjahn et al., 2005; Stence et al., 2001)—from the extension and retraction of their branches in response to physiological stimuli, to the migration of the entire cell body to the site of injury. Microglia motility has also been recently correlated with the ability of pruning synapses (Sipe et al., 2016). Therefore, motility represents an important feature to keep into account when trying to estimate microglial activity in physiological and pathological situations. Our gene expression profiling data and time‐lapse recordings respectively suggest a modulation by NGF of genes involved in cytoskeletal reorganization and of membrane dynamics.

We then focused on the possible consequences of NGF activity on microglial cells in pathological conditions, challenged with Aβ peptide, a well‐established neurodegenerative insult. We found that, while the effects of NGF on the inflammatory phenotype of naive microglia are slightly anti‐inflammatory, microglia treated with NGF become refractory to the potent inflammatory stimulus of Aβ.

We show that NGF is capable of enhancing specifically one type of endocytic process in microglial cells, the macropinocytosis, a mechanism of choice through which microglial cells clear sAβ (Mandrekar et al., 2009). Thus, as a further step, we demonstrated that, by enhancing macropinocytosis, NGF promotes sAβ clearance *in vitro* and, most remarkably, *ex vivo*. Increasing the uptake of sAβ is, however, might not actually provide a long term protection over the toxicity of the peptide, since internalized sAβ could be shed again into the extracellular space. In fact, microglia can release internalized Aβ and convert it in neurotoxic forms through the shedding of microvesicles (Joshi et al., 2014). Moreover, it is still not clear if microglial cells are actually able to digest sAβ efficiently (Lee et al., 2010; Majumdar et al., 2007; Mandrekar‐Colucci & Landreth, 2010), due to evidence suggesting that microglia near plaques are functionally impaired (Krabbe et al., 2013). Here, we showed that, in BV2 microglia, NGF not only increases Aβ uptake but enhances its degradation.

Alzheimer's disease has been described as a synaptopathy, entailing a dysfunction of synaptic function (Brose et al., 2010; Haass & Selkoe, 2007; Mucke & Selkoe, 2012). Synapse loss is indeed an early sign of AD and the process has been directly correlated with Aβ as the most likely culprit (Hong et al., 2016). High concentrations of Aβ or Aβ oligomers inhibit synaptic plasticity processes (Selkoe, 2008; Shankar et al., 2007; Walsh et al., 2002). Aβ has proven to be a key player in synaptic plasticity also at physiological concentrations: while short exposure with low concentrations of the peptide actually enhance synaptic plasticity, longer exposures lasting several hours reduce it (Koppensteiner et al., 2016). This underlines the importance of the homeostasis of Aβ levels and processing in the brain, and thus of microglia themselves as an important factor in its clearance. On their part, under physiological conditions microglial cells regulate dendritic spines, either pruning away superfluous spines during development (Schafer et al., 2012) or increasing spine density, as observed in the developing somatosensory cortex (Kettenmann, Kirchhoff, & Verkhratsky, 2013; Miyamoto et al., 2016). In relation to this microglia‐neuron communication, we demonstrate that NGF rescues the spine loss mediated by Aβ, an effect that is strictly dependent on microglia. Plasticity was also studied in vitro by evaluating the efficacy of chemical LTP in the presence of microglia. Interestingly, spine potentiation, measured as AMPAR intensity increase, is stronger in neurons cultured with microglia. While Aβ causes a dramatic loss of efficacy of chemical LTP in neuron‐microglia co‐cultures, NGF is able to fully rescue spine potentiation in these conditions. The effect is completely dependent on the presence of microglia in the cultures, since the Aβ‐induced LTP deficit is not rescued by NGF, when neurons are cultured in the absence of microglia. Thus, in this assay, NGF exerts its neuroprotective effects on neurons via microglia.

A further demonstration of NGF as a modulator of the microglia‐to‐neuron communication is provided by the observed stimulation by NGF of glutamatergic transmission in a microglia‐dependent manner. This demonstration adds one more line of evidence to the emerging theme of microglia as a modulator of neurotransmission (Cantaut‐Belarif et al., 2017; Marrone et al., 2017). Intranasal administration of an NGF variant was recently proven to be highly neuroprotective in an AD mouse model: 5×FAD mice chronically treated with the neurotrophin showed a dramatic reduction of the plaque load, with a clear evidence of the involvement of microglial cells in the clearance of Aβ (Capsoni et al., 2017). In that study, the neurotrophin, added to 5×FAD slices (which present synaptic transmission and LTP deficits), determined a TrkA‐dependent rescue of both synaptic transmission and synaptic plasticity deficits. Our results go in the direction of attributing those events to the action of NGF on microglial cell. By affecting microglial physiological activity, NGF is capable of influencing glutamatergic transmission. Indeed, along with TrkA expression in microglia, we found that tampering with NGF‐TrkA signaling affects negatively glutamatergic neurotransmission.

Thus, NGF‐activated microglia might result neuroprotective in Aβ pathology not only by lowering the amount of circulating Aβ—*per se* toxic to synapses and neurons—but also by aiding neurons in synaptic plasticity tasks.

Our data point toward these myeloid cells of the brain as the culprit for the severe neurodegeneration observed in anti‐NGF or anti‐TrkA mice (Capsoni et al., 2000; Capsoni et al., 2010), a conclusion that might be relevant also for human brain pathologies. Moreover, our results add an important element to the rationale for the therapeutic use of NGF in AD (Cattaneo & Calissano, 2012; Cattaneo et al., 2008; Eriksdotter Jönhagen et al., 1998; Eriksdotter‐Jönhagen et al., 2012; Eyjolfsdottir et al., 2016; Tuszynski et al., 2005, 2015). These broadly neuroprotective actions of NGF via microglia enlarge the spectrum of neurons that can be considered NGF targets—way beyond BFCN—thus extending the therapeutic potential of NGF and its derivatives (Capsoni et al., 2017). Future studies will be needed to investigate whether there are regional differences in the responsiveness to NGF of microglia from different brain regions and from different ages.

In any case, this demonstration of the broad influence of NGF on microglial cells vindicates the early and visionary view by Rita Levi‐Montalcini that considered NGF as a neurokine, a mediator of neuroimmune communication (Levi‐Montalcini, 1987; Levi‐Montalcini et al., 1996).

In conclusion, the evidence presented here corroborates the view that exploiting the innovative immunomodulatory and neuroprotective mechanisms displayed by NGF may be a viable clinical approach to ameliorate all hallmarks of AD pathology and, potentially, a spectrum of other neurodegenerative diseases.

## Supporting information

Additional Supporting Information may be found online in the supporting information tab for this article.

Supporting Information

Supporting Information
