# Supplementary figures and images for: NGF steers microglia toward a neuroprotective phenotype
Source: Glia. 2018 Feb 23;66(7):1395–416. doi: 10.1002/glia.23312 (PMC6001573; doi:10.1002/glia.23312)

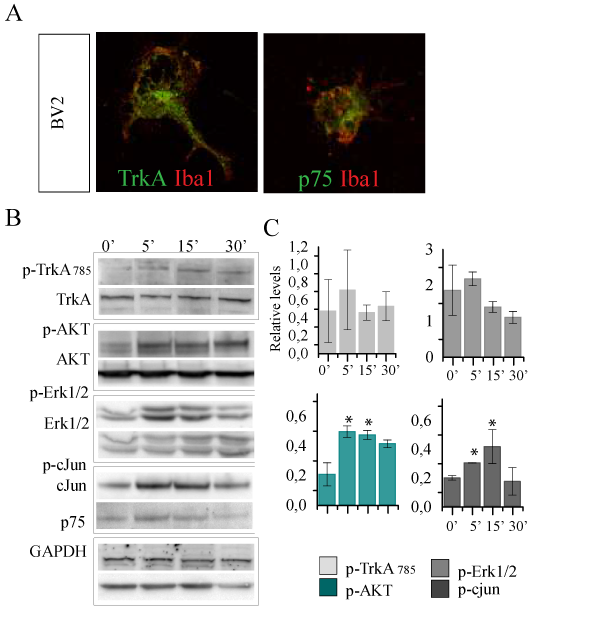

Supplement: Supplementary file 1 — Supporting Information [file GLIA-66-1395-s001.tif]
