# Supplementary material for: NGF steers microglia toward a neuroprotective phenotype
Source: Glia. 2018 Feb 23;66(7):1395–416. doi: 10.1002/glia.23312 (PMC6001573; doi:10.1002/glia.23312)
Supplement: Supplementary file 2 — Supporting Information [file GLIA-66-1395-s002.docx]

|  | NT |  | NGF |  | NGF Abeta |  | Abeta |  |
| --- | --- | --- | --- | --- | --- | --- | --- | --- |
|  | Mean | dev.st | Mean | dev.st | Mean | dev.st | Mean | dev.st |
|  |  |  |  |  |  |  |  |  |
| Fit-3 ligand | 0,01130403 | 0,00070111 | 0,00430909 | 0,02141182 | 0,01793163 | 0,00424088 | 0,03457731 | 0,03033075 |
|  |  |  |  |  |  |  |  |  |
| Il12 p70 | 0,07090061 | 0,00676126 | 0,02891708 | 0,0013701 | 0,03747002 | 0,00163407 | 0,12258251 | 0,01645122 |
|  |  |  |  |  |  |  |  |  |
| OPN | 0,26547454 | 0,01034522 | 0,14586039 | 0,01864768 | 0,14823767 | 0,02438268 | 0,34287222 |  |
|  |  |  |  |  |  |  |  |  |
| Fractalkine | 0,02964777 | 0,00285337 | 0,0332471 | 7,7147E-06 | 0,02656459 | 0,00115155 | -0,0055836 | 0,0273004 |
|  |  |  |  |  |  |  |  |  |
| Il-13 | 0,03702771 | 0,00844199 | 0,01901575 | 3,2447E-06 | 0,0116923 | 0,00393718 | 0,07398434 | 0,00527191 |
|  |  |  |  |  |  |  |  |  |
| OPG | 0,03073185 | 0,00137254 | 0,01098281 | 1,6528E-06 | 0,0067758 | 0,00314746 | 0,04433923 | 0,01559119 |
|  |  |  |  |  |  |  |  |  |
| Gelectin | 0,23508307 | 0,0174015 | 0,14267156 | 0,01414602 | 0,31537558 | 0,03169329 | 0,31067562 | 0,00221515 |
|  |  |  |  |  |  |  |  |  |
| Il15 | 0,01957125 | 0,0018311 | 0,00615646 | 0,00198344 | 0,01260577 | 0,00028013 | 0,07501362 | 0,00604433 |
|  |  |  |  |  |  |  |  |  |
| PRL | 0,02008799 | 0,00057669 | 0,01934244 | 0,00287985 | 0,01280913 |  | 0,05970095 | 0,02633333 |
|  |  |  |  |  |  |  |  |  |
| Gas6 | 0,03222121 | 0,00110719 | 0,02734119 | 0,00228126 | 0,03621601 | 0,00338743 | 0,08139218 | 0,00629362 |
|  |  |  |  |  |  |  |  |  |
| Il17 | 0,00390018 | 0,0030513 | 0,00447831 | 0,00097391 | 0,0019014 | 0,00477219 | 0,04302745 | 0,00361469 |
|  |  |  |  |  |  |  |  |  |
| ProMMP-9 | 0,02868488 | 0,00287028 | 0,0009402 | 0,00451829 | 0,00511127 | 0,00203722 | 0,05406598 |  |
|  |  |  |  |  |  |  |  |  |
| 6Ckine | 0,02277804 | 0,00273269 | 0,00860859 | 0,00021597 | 0,00079413 | 0,00114315 | 0,05670951 | 0,015758 |
|  |  |  |  |  |  |  |  |  |
| G-CSF | 0,00013579 | 0,00881682 | 0,00236739 | 0,00183494 | -0,006329 | 0,00344696 | 0,04242511 | 0,0069324 |
|  |  |  |  |  |  |  |  |  |
| Il-17BR | 0,01860697 | 0,00301985 | 0,01590349 | 0,0004856 | 0,01133839 | 0,00093469 | 0,04692791 | 0,00731534 |
|  |  |  |  |  |  |  |  |  |
| RANTES | 0,01728468 | 0,00199895 | 0,01268114 | 0,00326496 | 0,00789726 | 0,00973323 | 0,05383023 | 0,01901124 |
|  |  |  |  |  |  |  |  |  |
| ALK-1 | 0,03877558 | 0,00020795 | 0,02004928 | 0,00026338 | 0,04393781 | 0,00224265 | 0,06279269 | 0,01243722 |
|  |  |  |  |  |  |  |  |  |
| GITR | 0,03403176 | 0,0033709 | 0,01784711 | 0,00129001 | 0,03679325 | 0,00206407 | 0,06036741 | 0,02585297 |
|  |  |  |  |  |  |  |  |  |
| IL-17E | 0,08285036 | 0,008076 | 0,03777696 | 0,00423391 | 0,0455307 | 0,0143077 | 0,09048751 | 0,01556152 |
|  |  |  |  |  |  |  |  |  |
| SCF | 0,04100392 | 0,00063703 | 0,01477864 | 0,00317921 | 0,01177136 | 0,00884056 | 0,07867267 | 0,01209378 |
|  |  |  |  |  |  |  |  |  |
| AREG | 0,01711803 | 0,00693226 | 0,01517205 | 0,00230768 | 0,01544144 | 0,00086302 | 0,03629532 | 0,02478152 |
|  |  |  |  |  |  |  |  |  |
| GITR ligand | 0,02094914 | 0,00090218 | 0,01074903 | 0,0031003 | 0,01020835 | 0,00079929 | 0,06298981 | 0,00611372 |
|  |  |  |  |  |  |  |  |  |
| Il-17F | 0,01021231 | 0,00683182 | 0,0137532 | 0,00241218 | -0,0003105 | 0,00461112 | 0,06161507 | 0,01259379 |
|  |  |  |  |  |  |  |  |  |
| sTNFRI | 0,02463765 | 0,0050418 | 0,00935604 | 0,00287384 | 0,00187367 | 0,00620737 | 0,09969152 | 0,01261037 |
|  |  |  |  |  |  |  |  |  |
| Axl | 0,03071851 | 7,4494E-05 | 0,00985462 | 0,00063351 | 0,02316258 | 0,00102363 | 0,03671676 | 0,02993308 |
|  |  |  |  |  |  |  |  |  |
| Granzyme B | 0,04832521 | 0,00567608 | 0,02122159 | 0,00132563 | 0,04529812 | 0,00270882 | 0,09760228 | 0,00701836 |
|  |  |  |  |  |  |  |  |  |
| Il-20 | 0,03685676 | 0,00893573 | 0,01780091 | 0,00250112 | 0,02698139 | 0,00656757 | 0,09338083 | 0,00857488 |
|  |  |  |  |  |  |  |  |  |
| sTNRII | 0,09060209 | 0,00953168 | 0,04335002 | 0,00445742 | 0,04776899 | 0,01582085 | 0,14499475 | 0,02373504 |
|  |  |  |  |  |  |  |  |  |
| BLC | 0,03734904 | 0,00282369 | 0,01897097 | 0,00103347 | 0,04698097 | 0,00388512 | 0,05502897 | 0,02222477 |
|  |  |  |  |  |  |  |  |  |
| HAI-1 | 0,01084107 | 0,00437057 | 0,00829049 | 0,002549 | 0,0166697 | 0,00774176 | 0,08891768 | 0,00719479 |
|  |  |  |  |  |  |  |  |  |
| IL-21 | 0,05908789 | 0,000228 | 0,03371758 | 0,00472835 | 0,03254048 | 0,0031052 | 0,10879972 | 0,00712704 |
|  |  |  |  |  |  |  |  |  |
| TACI | 0,05544997 | 0,00195119 | 0,02693376 | 0,00264018 | 0,02320913 | 0,00442203 | 0,13440892 | 0,01905401 |
|  |  |  |  |  |  |  |  |  |
| CT-1 | 0,03366206 | 0,00327223 | 0,00915321 | 0,00218831 | 0,01329823 | 0,00591931 | 0,05546069 | 0,02581654 |
|  |  |  |  |  |  |  |  |  |
| HGF | 0,04795787 | 0,00337385 | 0,02451225 | 0,00420206 | 0,03109649 | 0,00596763 | 0,10527974 | 0,00548313 |
|  |  |  |  |  |  |  |  |  |
| I-Tac | 0,03607011 | 0,00417205 | 0,01688923 | 0,0018235 | 0,0183471 | 0,00056072 | 0,09996345 | 0,00117031 |
|  |  |  |  |  |  |  |  |  |
| TARC | 0,02978731 | 0,00603676 | 0,01626888 | 0,00295911 | 0,01570572 | 0,0030347 | 0,11757072 | 0,01955116 |
|  |  |  |  |  |  |  |  |  |
| CD27 | 0,02356219 | 0,00300786 | 0,0104643 | 0,00254051 | -0,001031 | 0,00593542 | 0,03501611 | 0,02443173 |
|  |  |  |  |  |  |  |  |  |
| IFN gamma | 0,00261388 | 0,00616766 | 0,00314537 | 0,00173633 | -0,0058516 | 0,00311314 | 0,07899251 | 0,0142461 |
|  |  |  |  |  |  |  |  |  |
| JAM-A | 0,03579492 | 0,00050593 | 0,01842426 | 0,00309016 | 0,03828248 | 0,00972273 | 0,1223385 | 0,01168935 |
|  |  |  |  |  |  |  |  |  |
| TNF alpha | -0,0075178 | 0,00764024 | 0,00199183 | 0,0048371 | 0,00334853 | 0,0045768 | 0,03753952 | 0,02986533 |
|  |  |  |  |  |  |  |  |  |
| CD27 ligand | 0,02299041 | 0,00248464 | 0,01263694 | 0,00158253 | 0,03734127 | 0,01159491 | 0,10032526 | 0,00674594 |
|  |  |  |  |  |  |  |  |  |
| IGFBP 5 | 0,04367756 | 0,00995781 | 0,01490691 | 0,00119412 | 0,04642486 | 0,01714048 | 0,10088665 | 0,00109601 |
|  |  |  |  |  |  |  |  |  |
| KC | 0,01453139 | 0,00515561 | 0,0068995 | 0,00338031 | 0,00895269 | 0,00443837 | 0,12338297 | 0,0080763 |
|  |  |  |  |  |  |  |  |  |
| TPO | 0,0630976 | 0,003479 | 0,0225296 | 0,00330895 | 0,02085611 | 0,00074887 |  |  |
|  |  |  |  |  |  |  |  |  |
| CD30 | 0,02518275 | 0,00429136 | 0,00727481 | 0,00222441 | -0,0029052 | 0,03217954 | 0,05817426 | 0,01665098 |
|  |  |  |  |  |  |  |  |  |
| IGFBP 6 | 0,02945263 | 0,00239855 | 0,01248715 | 0,00020983 | 0,02470364 | 0,00660235 | 0,09929222 | 0,00292474 |
|  |  |  |  |  |  |  |  |  |
| Leptin | 0,03981088 | 0,01292833 | 0,01904586 | 0,00170437 | 0,0081338 | 0,01171069 | 0,11762904 | 0,00762725 |
|  |  |  |  |  |  |  |  |  |
| Trance | 0,02490075 | 0,0051159 | 0,00719874 | 0,00342891 | -0,033309 | 0,00219246 | 0,10080835 | 4,1343E-05 |
|  |  |  |  |  |  |  |  |  |
| CD30 ligand | 0,02900149 | 0,00036697 | 0,01730716 | 0,00286582 | 0,03822322 | 0,00142094 | 0,08427248 | 0,02413639 |
|  |  |  |  |  |  |  |  |  |
| IGF-2 | 0,04792716 | 9,7491E-05 | 0,02462634 | 0,00306031 | 0,05347863 | 0,00200921 | 0,14390932 | 0,00197732 |
|  |  |  |  |  |  |  |  |  |
| LeptinR | 0,03770873 | 0,00530695 | 0,00926371 | 0,0016586 | 0,01794484 | 0,00235096 | 0,10363409 | 0,00055118 |
|  |  |  |  |  |  |  |  |  |
| TROY | 0,09011411 | 0,0063996 | 0,04628855 | 0,00182043 | 0,08409428 | 0,06113576 | 0,15979393 | 0,00953376 |
|  |  |  |  |  |  |  |  |  |
| CD36 | 0,09569003 | 0,02433533 | 0,03059615 | 0,00109764 | 0,08426479 | 0,0100339 | 0,10108854 | 0,02409996 |
|  |  |  |  |  |  |  |  |  |
| IL1 aplha | 0,13666185 | 0,02846689 | 0,058044 | 0,00310466 | 0,11191371 | 0,00165111 | 0,17573108 | 0,0011965 |
|  |  |  |  |  |  |  |  |  |
| L Selectin | 0,0357396 | 0,00188259 | 0,01863494 | 0,00178304 | 0,03811262 | 0,00189809 | 0,11687865 | 0,00325815 |
|  |  |  |  |  |  |  |  |  |
| TWEAK R | 0,09681108 | 0,01281728 | 0,05414134 | 0,00491601 | 0,05458557 | 0,01078511 | 0,15463756 |  |
|  |  |  |  |  |  |  |  |  |
| CTLA-4 | -0,006902 | 0,01881649 | 0,01926137 | 0,00246137 | 0,04774935 | 0,00176807 | 0,05512232 | 0,02562824 |
|  |  |  |  |  |  |  |  |  |
| IL-1Beta | 0,01771511 | 0,02043315 | 0,00611935 | 0,00108266 | 0,0163914 | 0,00047948 | 0,09282176 | 0,00130723 |
|  |  |  |  |  |  |  |  |  |
| Lungkine | 0,11727281 | 0,0057722 | 0,05266161 | 0,0037134 | 0,09948901 | 0,00843881 | 0,14190301 | 0,01249309 |
|  |  |  |  |  |  |  |  |  |
| VCAM-1 | 0,07443999 | 0,01291536 | 0,03774477 | 0,00237196 | 0,04682102 | 0,01464268 | 0,15092482 | 0,00823799 |
|  |  |  |  |  |  |  |  |  |
| CXCL16 | -0,0296311 | 0,00892413 | 0,01431192 | 0,00138072 | 0,06184534 | 0,01138855 | 0,04621007 | 0,02285884 |
|  |  |  |  |  |  |  |  |  |
| IL-1ra | 0,0538037 | 0,0131868 | 0,02814494 | 0,00250548 | 0,11541162 | 0,06901852 | 0,09353496 | 0,00529012 |
|  |  |  |  |  |  |  |  |  |
| Mad CAM-1 | 0,01100619 | 0,00207128 | 0,00513795 | 0,00638483 | 0,01413742 | 0,00557102 |  |  |
|  |  |  |  |  |  |  |  |  |
| VEGF | 0,05086154 | 0,01454597 | 0,0193296 | 0,00359958 | 0,02532115 | 0,01036422 | 0,13321494 | 0,00338853 |
|  |  |  |  |  |  |  |  |  |
| Decorin | 0,10570832 | 0,00638014 | 0,02890741 | 0,0008983 | 0,15077111 | 0,01631224 | 0,0593555 | 0,02773635 |
|  |  |  |  |  |  |  |  |  |
| Il-2 | 0,04735662 | 0,003274 | 0,01615387 | 0,00019473 | 0,03431544 | 0,00570875 | 0,07187296 | 0,01634561 |
|  |  |  |  |  |  |  |  |  |
| MCP-1 | 0,06717485 | 0,00385796 | 0,02351316 | 0,00312082 | 0,04119705 | 0,00200034 | 0,14117013 | 0,0028044 |
|  |  |  |  |  |  |  |  |  |
| VEGF R1 | 0,10924144 | 0,01646708 | 0,03149498 | 0,00300995 | 0,03976064 | 0,00622044 | 0,14798026 | 0,01444565 |
|  |  |  |  |  |  |  |  |  |
| DKK-1 | 0,05474824 | 0,00382297 | 0,01830975 | 0,00017692 | 0,06504432 | 0,02241386 | 0,06451071 | 0,00872675 |
|  |  |  |  |  |  |  |  |  |
| IL-2 R alpha | 0,02817453 | 0,00173302 | 0,01821942 | 0,00686265 | 0,03730231 | 0,00082871 | 0,10505325 | 0,01653882 |
|  |  |  |  |  |  |  |  |  |
| MDC | 0,06557764 | 0,00923135 | 0,03659297 | 0,00146446 | 0,05329442 | 0,00110487 | 0,1761968 | 0,00371723 |
|  |  |  |  |  |  |  |  |  |
| VEGF R3 | 0,02581388 | 0,00898251 | 0,01628298 | 0,004887 | 0,00513092 | 0,00897572 | 0,12585153 | 0,02100841 |
|  |  |  |  |  |  |  |  |  |
| E Cadherin | 0,01400964 | 0,00663684 | 0,01789966 | 0,00059576 | 0,02525248 | 0,00073533 | 0,03094647 | 0,01761334 |
|  |  |  |  |  |  |  |  |  |
| Il-3 | 0,10379603 | 0,00277475 | 0,04327212 | 0,00809923 | 0,12078453 | 0,00720532 | 0,12008819 | 0,02096564 |
|  |  |  |  |  |  |  |  |  |
| MFG E8 | 0,05663162 | 0,0161699 | 0,00917572 | 0,00299391 | 0,02597316 | 0,00720159 | 0,15026545 | 0,0045093 |
|  |  |  |  |  |  |  |  |  |
| VEGFD | 0,06511413 | 0,01341146 | 0,0133291 | 0,00136232 | 0,03188534 | 0,00017111 | 0,12264315 | 0,01319962 |
|  |  |  |  |  |  |  |  |  |
| EGF | 0,02467907 | 0,0011009 | 0,01333611 | 0,0011677 | 0,0399021 | 0,00568797 | 0,02658377 | 0,01626968 |
|  |  |  |  |  |  |  |  |  |
| IL-4 | 0,05915419 | 0,00591293 | 0,01017381 | 0,01928283 | 0,01192884 | 0,00547811 | 0,10949295 | 0,01571993 |
|  |  |  |  |  |  |  |  |  |
| MIG | 0,04119961 | 0,00208996 | 0,01270224 | 0,00018471 | 0,06594674 | 0,01351214 | 0,12769083 | 0,01431712 |
|  |  |  |  |  |  |  |  |  |
| Eotaxin1 | 0,03110016 | 0,00690553 | 0,01913118 | 0,00073329 | 0,0399021 | 0,00568797 | 0,04256636 | 0,02272355 |
|  |  |  |  |  |  |  |  |  |
| MIP-1 alpha | 0,07932766 | 0,00652559 | 0,04806086 | 0,00148427 | 0,06711046 | 0,01515788 | 0,15729976 | 0,00957838 |
|  |  |  |  |  |  |  |  |  |
| Eotaxin 2 | 0,03196881 | 0,00221143 | 0,02882826 | 0,00259689 | 0,03726088 | 0,00641186 | 0,03448989 | 0,02725578 |
|  |  |  |  |  |  |  |  |  |
| MIP-1Gamma | 0,20048759 | 0,01471225 | 0,10423814 | 0,00116959 | 0,25192679 | 0,04522107 | 0,23493491 | 0,01789046 |
|  |  |  |  |  |  |  |  |  |
| Epigen | 0,0057124 | 0,00655173 | 0,00231168 | 0,00014354 | -0,0012573 | 0,0005096 | 0,0262276 | 0,03001392 |
|  |  |  |  |  |  |  |  |  |
| Il-9 | 0,0183019 | 0,00637169 | 0,01058657 | 0,00547297 | 0,01663603 | 0,00254822 | 0,0935804 | 0,01802759 |
|  |  |  |  |  |  |  |  |  |
| MIP2 | 0,07696965 | 0,00579834 | 0,03696412 | 0,00139581 | 0,03997358 | 0,0047311 | 0,15930954 | 0,01503183 |
|  |  |  |  |  |  |  |  |  |
| E Selectin |  |  |  |  |  |  | 0,02332154 | 0,02922185 |
|  |  |  |  |  |  |  |  |  |
| MIP-3 alpha | 0,02658775 |  | 0,02076871 | 0,00587187 | 0,00682565 | 0,00350555 | 0,12636747 | 0,00306678 |
|  |  |  |  |  |  |  |  |  |
| Fas Ligand | 0,02557774 | 0,00395093 | 0,01727547 | 0,0019109 | 0,04158297 | 0,04158297 | 0,04725484 | 0,0397325 |
|  |  |  |  |  |  |  |  |  |
| IL-11 | 0,06967518 | 0,00732831 | 0,03393651 | 0,00781214 | 0,0921812 | 0,0921812 | 0,1653577 | 0,02105631 |
|  |  |  |  |  |  |  |  |  |
| MIP-3beta | 0,02440555 | 0,01046315 | 0,00065605 |  | 0,03940129 | 0,03940129 | 0,15684692 | 0,02293784 |
|  |  |  |  |  |  |  |  |  |
| Fcg RIIB | 0,06963363 | 0,00012127 | 0,05635802 | 0,00189509 | 0,14713982 | 0,00678537 | 0,11548032 | 0,03851103 |
|  |  |  |  |  |  |  |  |  |
| IL-12 p40 | 0,02824166 | 0,0015398 | 0,0110829 | 0,00104385 | 0,0238245 | 0,00213993 | 0,10666967 | 0,01871917 |
|  |  |  |  |  |  |  |  |  |
| MMP-2 | 0,05314533 | 0,01319839 | -0,016358 | 0,0165365 | 0,05233984 | 0,00071993 | 0,14108141 | 0,024189 |
